# Pregnancy dedifferentiates memory CD8^+^ T cells into hypofunctional cells with exhaustion-enriched programs

**DOI:** 10.1172/jci.insight.176381

**Published:** 2024-05-21

**Authors:** Jared M. Pollard, Grace Hynes, Dengping Yin, Malay Mandal, Fotini Gounari, Maria-Luisa Alegre, Anita S. Chong

**Affiliations:** 1 Section of Transplantation, Department of Surgery, and; 2 Section of Rheumatology, Department of Medicine, University of Chicago, Chicago, Illinois, USA.; 3 Department of Immunology, Mayo Clinic, Phoenix, Arizona, USA.

**Keywords:** Immunology, Transplantation, Bioinformatics, T cells

## Abstract

Alloreactive memory, unlike naive, CD8^+^ T cells resist transplantation tolerance protocols and are a critical barrier to long-term graft acceptance in the clinic. We here show that semiallogeneic pregnancy successfully reprogrammed memory fetus/graft-specific CD8^+^ T cells (T_FGS_) toward hypofunction. Female C57BL/6 mice harboring memory CD8^+^ T cells generated by the rejection of BALB/c skin grafts and then mated with BALB/c males achieved rates of pregnancy comparable with naive controls. Postpartum CD8^+^ T_FGS_ from skin-sensitized dams upregulated expression of T cell exhaustion (T_EX_) markers (Tox, Eomes, PD-1, TIGIT, and Lag3). Transcriptional analysis corroborated an enrichment of canonical T_EX_ genes in postpartum memory T_FGS_ and revealed a downregulation of a subset of memory-associated transcripts. Strikingly, pregnancy induced extensive epigenetic modifications of exhaustion- and memory-associated genes in memory T_FGS_, whereas minimal epigenetic modifications were observed in naive T_FGS_. Finally, postpartum memory T_FGS_ durably expressed the exhaustion-enriched phenotype, and their susceptibility to transplantation tolerance was significantly restored compared with memory T_FGS_. These findings advance the concept of pregnancy as an epigenetic modulator inducing hypofunction in memory CD8^+^ T cells that has relevance not only for pregnancy and transplantation tolerance, but also for tumor immunity and chronic infections.

## Abstract 2

Pregnancy overrides T cell memory, resulting in improved transplantation outcomes in mice.

## Introduction

Mammalian pregnancy has long been recognized as a model of spontaneous alloantigen-specific tolerance, whereby the maternal adaptive immune system must rapidly regulate responses toward the semiallogeneic fetus to preserve fetal viability (1, 2). Maternal T cell tolerance is characterized by the upregulation of coinhibitory markers and inhibition of proinflammatory cytokine production in CD4^+^FoxP3^–^ conventional T cells (T_convs_), as well as the expansion of fetus-specific CD4^+^FoxP3^+^ Tregs that are protective in primary and secondary pregnancies (3–5). Notably, while pregnancy efficiently tolerizes naive fetus-specific T cells, it simultaneously induces humoral sensitization. We showed that pregnancy induced a state of T cell tolerance that was sufficient to mediate the spontaneous acceptance of subsequently transplanted offspring-matched heart grafts but only if B cells and fetus-specific antibodies are absent (3). Subsequently, Lewis et al. reported that naive OVA-reactive TCR-transgenic OT-I CD8^+^ T cells acquired an exhausted transcriptional signature after pregnancy with OVA-expressing progeny (6), while Kinder et al. showed that endogenous OVA-reactive CD8^+^ T cell expression of PD-1 and Lag-3 acquired during primary pregnancy protected against fetal wastage in a secondary pregnancy (7).

In contrast to pregnancy, semiallogeneic organ transplants stimulate alloreactive CD4^+^ and CD8^+^ T cells that mediate graft rejection, with CD4^+^ T cells promoting B cell and CD8^+^ T cell responses as well as secreting proinflammatory cytokines and chemokines; furthermore, CD8^+^ T cells play proinflammatory and cytotoxic roles (8–11). Alloreactive memory T cells are generated by exposure to alloantigen following transplantation or blood transfusion, or through heterologous immunity, wherein T cells primed by infections or environmental antigens cross-react with donor alloantigens (12–15). As a result, most humans harbor memory alloreactive T cells, and their frequency increases with age (8). Importantly, memory T cells antagonize therapies that successfully induce transplantation tolerance in naive mice by resisting the induction of cell-intrinsic hypofunction achieved in naive T cells (16–18). Indeed, we recently reported that the presence of memory T cells sensitized to a single donor antigen mediated linked sensitization and was sufficient to prevent costimulation blockade–induced transplantation tolerance to multiple-antigen mismatch allografts (19). Thus, the potent barrier posed by alloreactive memory T cells to transplantation tolerance underscores the critical need to identify mechanisms for tolerizing memory T cell responses (20–23).

The role of memory CD8^+^ T cells in mediating allograft rejection, inducing spontaneous abortions, and antagonizing tolerance prompted this proof-of-principle study to test whether pregnancy can successfully program hypofunction into memory fetus/graft-specific CD8^+^ T cells (T_FGS_) (8–11, 24, 25). We show that, despite the presence of rejection-induced memory CD4^+^, CD8^+^, and B cell responses, sensitized female mice consistently achieved spontaneous tolerance toward the semiallogeneic fetus, achieving pregnancy success rates comparable with those of naive mice. We then used high-dimensional multiomics approaches to show that pregnancy dedifferentiates memory CD8^+^ T cells into hypofunctional cells with an exhaustion phenotype and reduced expression of a subset of memory genes. The pregnancy-programmed hypofunctional phenotype in memory T_FGS_ was resistant to NFAT inhibition, associated with extensive epigenetic remodeling, persisted postpartum, and manifested as restored susceptibility to costimulation blockade–mediated transplantation tolerance. Taken together, our findings highlight the evolutionary robustness of mammalian pregnancy in constraining fully established allogeneic memory responses and introduce a potentially novel hypothesis that successful reprogramming of memory CD8^+^ T cells toward hypofunction requires the epigenetic imprinting of exhaustion circuits and reduced expression of a subset of memory genes. The conceptual foundation provided here brings us closer to understanding and therapeutically harnessing mechanisms of antigen-specific T cell hypofunction to substantially reduce the barrier that memory CD8^+^ T cells pose to transplantation tolerance.

## Results

### Pregnancy successfully constrains immunological memory.

**Figure 1:**
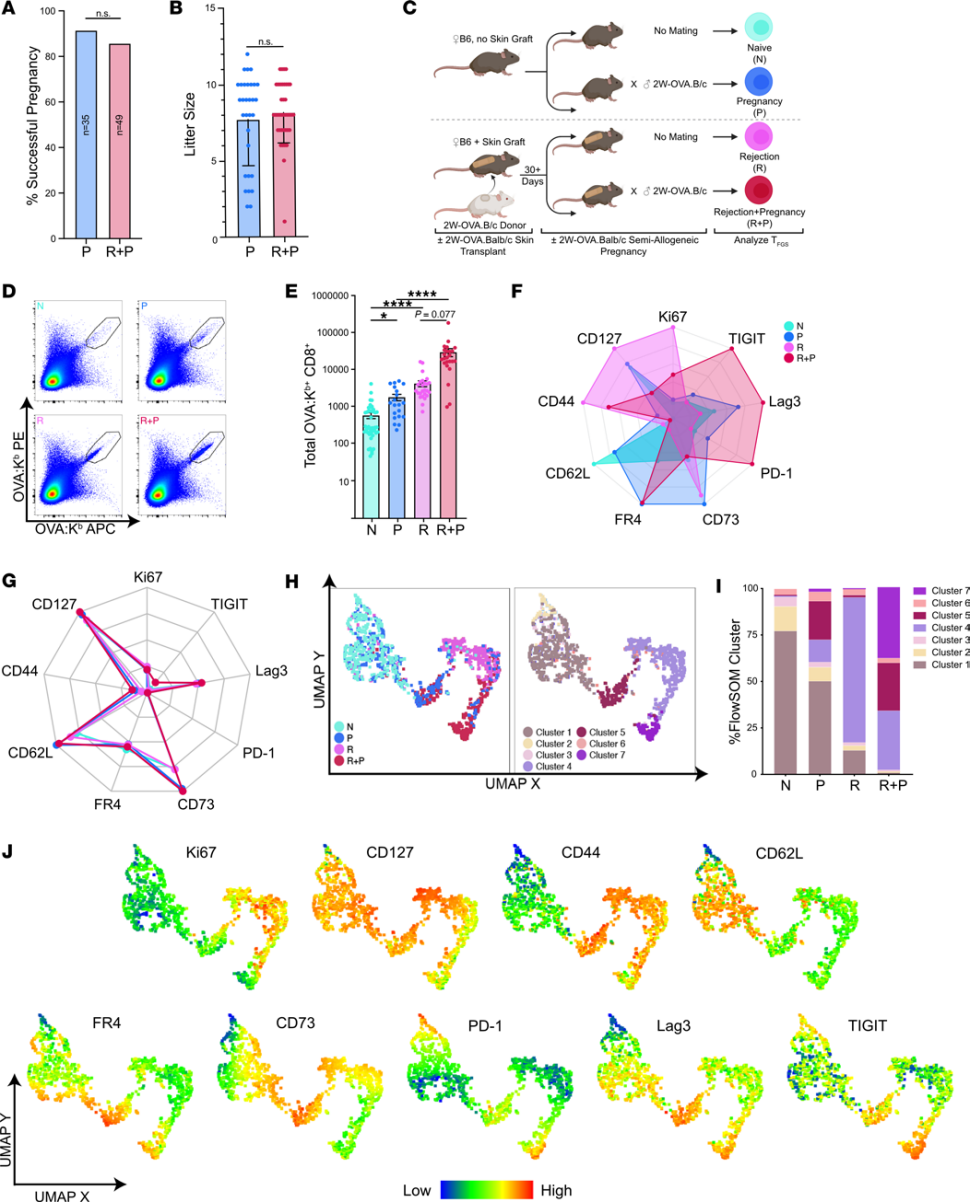
Pregnancy induces a hypofunctional phenotype in memory OVA-specific T_FGS_. (**A**) Bar graph showing percentage of P vs. R + P mice achieving successful full-term pregnancies; *n* = 35–49 mated mice per group. Additionally, there was a 100% success rate in sensitized mice subjected to a second pregnancy (*n* = 12). *P* values were determined by χ^2^ test of independence. (**B**) Bar graph showing number of viable pups at birth (litter sizes) of P vs. R + P mice female mice achieving successful full-term pregnancies; *n* = 31–39 per group. Each dot indicates individual mice. (**C**) Experimental design. Female B6 mice were mated with transgenic 2W-OVA.B/c mice, with or without sensitization to 2W-OVA.B/c via skin graft 30 days prior (R+P and P, respectively). Unmated mice with or without skin graft rejection were included as controls (naive [N] and rejection [R], respectively). (**D**) Representative pseudocolor plots showing OVA:K^b^-specific CD8^+^ T cells (T_FGS_). Each dot indicates an individual mouse. (**E**) Normalized total recovery of T_FGS_ at postpartum days 0–3. Data acquired from 2 or more biologically independent experiments; *n* = 20–38 per group. *P* values were determined by Kruskal-Wallis 1-way ANOVA with Dunn’s post hoc test. (**F** and **G**) Radar plot showing phenotypic profile of T_FGS_ (**F**) or non-T_FGS_ (**G**) based on markers of activation, memory, and coinhibition. Data are normalized to the highest and lowest MFI for each marker expressed by T_FGS_ or non-T_FGS_ from all 4 experimental groups. Symbols color coded as in **D**. Expression is represented as normalized percentage of the highest/lowest-expressing group (based on all OVA^+^T_FGS_ and non- T_FGS_) for each marker. (**H**) UMAP with experimental groups (left) and FlowSOM clustering (right) reveals distinct phenotypic subsets in T_FGS_. (**I**) Stacked bar graph showing FlowSOM cluster distributions for each experimental group. (**J**) UMAP with heatmap overlays to show expression of each phenotypic marker on T_FGS_ at single-cell resolution. Data represent mean ± SEM. Gating strategy, statistical analysis, and representative histograms of this flow data set are in Supplemental Figure 1, A–C. *P < 0.05; ****P < 0.0001.

To test whether semiallogeneic pregnancy is possible in females harboring immunological memory to paternal antigens, we sensitized female C57BL/6 (B6, H-2^b^) mice with skins transplants (skinTx) from fully mismatched male BALB/c mice. Female B6 mice rejected BALB/c skin grafts within 10 days (data not shown), and at day ≥30 after transplantation, they were mated with BALB/c males (rejection+pregnancy [R+P]). The rates of successful pregnancy, including multiple successive pregnancies, were comparable between R+P and control naive mice mated with BALB/c males (pregnancy only [P]), and no differences in resulting viable pups were observed (Figure 1, A and B). Thus, pregnancy is able to constrain memory immune responses elicited by the rejection of fully mismatched skin allografts to permit successful tolerance of the semiallogeneic fetus.

### Pregnancy induces the expression of coinhibitory molecules in memory T_FGS_.

To gain insights into pregnancy-imposed hypofunction, we tracked a tracer population of endogenous, polyclonal fetus–reactive CD8^+^ T cells that recognize the model 2W-OVA antigen expressed by the sensitizing skin and fetus. We sensitized B6 females with 2W-OVA.BALB/c (H-2^d^) skins and then mated them with 2W-OVA.BALB/c (H-2^d^) males (Figure 1C). OVA-specific CD8^+^ T cells were identified by flow cytometry using double fluorophore–labeled OVA:K^b^ (OVA257-264 peptide presented on MHC Class I [Kb]) tetramers; henceforth, these fetus- and graft-specific T cells are referred to as T_FGS_ (26, 27). We note that OVA expressed by the skin or F1 fetus is cross-presented by recipient/dam antigen presenting cells (APCs), although it is possible that maternal APCs are cross-decorated with OVA:K^b^ complexes from F1 cells or that microchimeric fetal cells are presenting OVA:K^b^ to maternal T cells (7, 28).

We designed a 19-color spectral flow cytometry panel to probe the expression of activation, memory, coinhibitory, and anergy markers by fetus-specific (OVA-specific) T_FGS_ from naive (N), P, skin rejection (R) and R+P groups (Supplemental Table 1; supplemental material available online with this article; https://doi.org/10.1172/jci.insight.176381DS1). OVA-specific CD8^+^ T cells were analyzed on day 30+ after skinTx for R mice or at postpartum days 0–3 for P and R+P mice. We observed a significant increase in T_FGS_ recovery from R+P mice compared with P mice (Figure 1, D and E). Despite this expansion, R+P T_FGS_ displayed elevated expression of multiple coinhibitory markers compared with R T_FGS_, including PD-1, LAG3, TIGIT, and FR4 (Figure 1F and Supplemental Figure 1, A and B). In contrast, P T_FGS_ preferentially upregulated both anergy markers, CD73 and FR4, as well as LAG3 and PD-1, compared with N T_FGS_. Finally, only postpartum CD8^+^ T_FGS_ exhibited this phenotype in response to pregnancy, as the non–OVA-specific CD8^+^ T cells from P, R, and R+P all resembled N T cells, thus confirming that the pregnancy-induced phenotype in T_FGS_ was driven by antigen recognition (Figure 1G).

To visualize T_FGS_ phenotypes at single-cell resolution, we used uniform manifold approximation and projection (UMAP) dimensionality reduction and FlowSOM clustering to identify 4 major and 3 minor cell subsets (Figure 1H). As anticipated, N and R T_FGS_ were largely homogenous, with > 75% of these cells mapping to Cluster 1 (C1) or C4, respectively (Figure 1I). In contrast, the effect of pregnancy on T_FGS_ was heterogeneous, with ~50% of P and ~25% of R+P T_FGS_ remaining phenotypically similar to N or R T_FGS_, respectively. Notably, C5 was identified as a shared cluster induced by pregnancy, comprising ~25% of both P and R+P T_FGS_ and defined by elevated expression of multiple coinhibitory markers and reduced expression of the proliferation marker Ki67 (Figure 1J and Supplemental Figure 1C). C7 was unique to R+P T_FGS_ and was similar to C5 except for reduced CD73 expression. Collectively, these observations support a hypothesis that the encounter of alloantigen during pregnancy programs hypofunction in memory T_FGS_ through the induction of higher levels of coinhibitory exhaustion markers and lower levels of anergy markers compared with postpartum N T_FGS_.

### Pregnancy induces both distinct and shared transcriptional modifications in N and memory T_FGS_.

**Figure 2:**
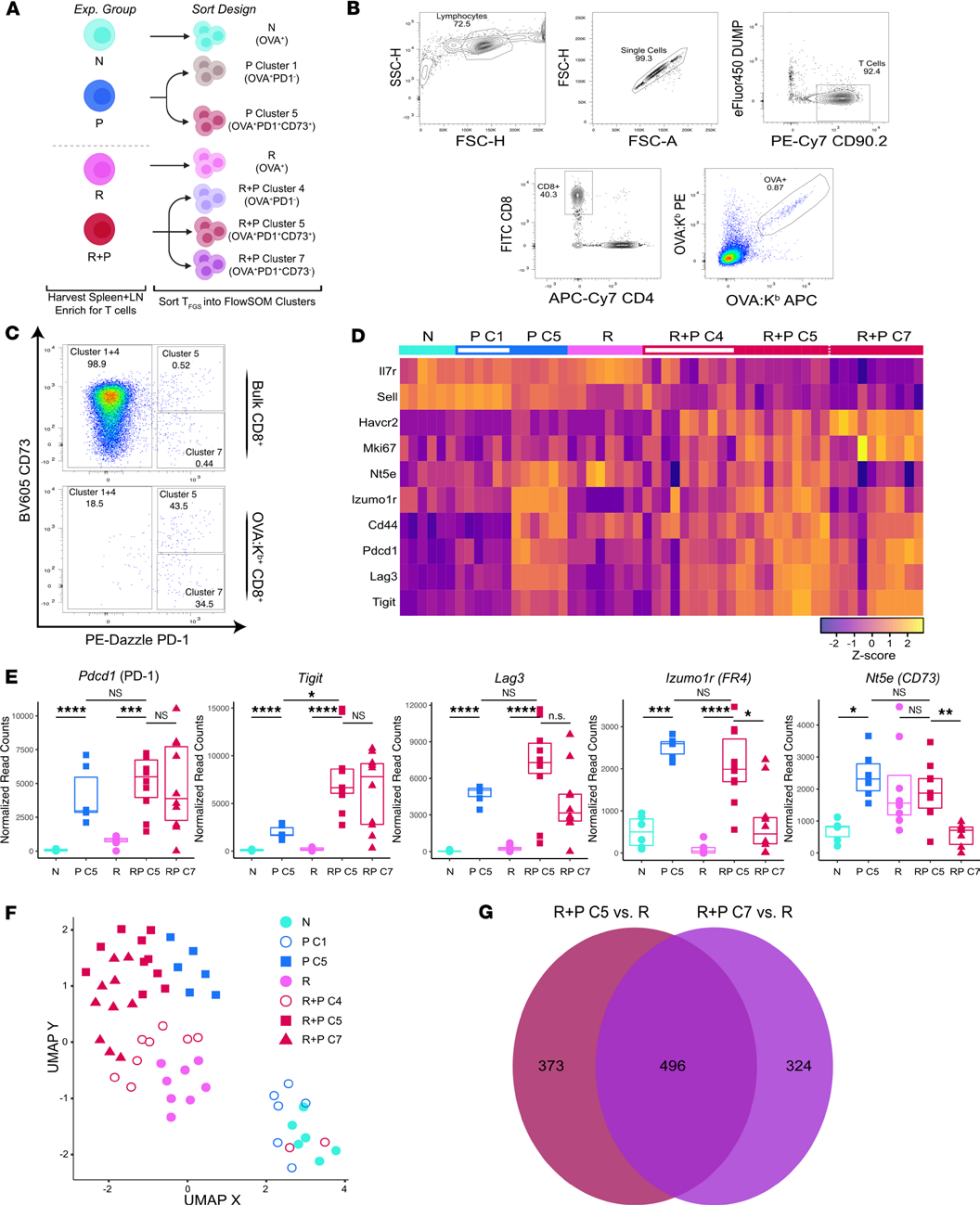
Pregnancy induces broad transcriptional modification in memory OVA-specific T_FGS_. (**A**) Sorting strategy for OVA:K^b^-specific T_FGS_ from each experimental group into their most prevalent phenotypic subsets as defined by FlowSOM (in Figure 1I). T_FGS_ were acquired and flow sorted from 2 biologically independent experiments; *n* = 3–5 per group, with technical replicates for each biological sample. (**B**) Gating strategy for each cluster of OVA:K^b^-specific T_FGS_. (**C**) Representative plots showing the distributions of C1+C4, C5, and C7 within bulk or OVA:K^b^-specific R+P CD8^+^ T cells. Percentage of each cell cluster is comparable to the distribution of our FlowSOM analysis in Figure 1H for the experimental groups. (**D** and **E**) Row-normalized RNA-Seq expression and box plots of normalized RNA-Seq read counts for key exhaustion and anergy markers corresponding to Figure 1I. Each dot in box plots or UMAP, and each column in the heatmap, indicates an individual mouse. *P* values (in **E**) were determined by Kruskal-Wallis 1-way ANOVA with Dunn’s post hoc test. *P < 0.05; ***P < 0.001; ****P < 0.0001. (**F**) UMAP comparing all T_FGS_ subsets analyzed by RNA-Seq. (**G**) Venn diagram of DEGs unique to R+P C7 T_FGS_, R+P C5 T_FGS_, and shared between both subsets.

We next tested the hypothesis that the difference in phenotypic markers induced by pregnancy was indicative of a broader set of transcriptional modifications induced in memory vs. N T_FGS_. We performed genome-wide transcriptional profiling of flow-sorted T_FGS_ subsets to account for the heterogeneity among pregnancy-modified T_FGS_ while retaining the advantageous sequencing depth of bulk RNA-Seq. We sorted OVA-specific T_FGS_ into the 4 predominant phenotypic subsets as illustrated in Figure 1, G and H: C1 (naive-like phenotype), C4 (rejection-like phenotype), C5 (shared by P and R+P), and C7 (unique to R+P) (Figure 2A). The proportions of each cluster in this panel were consistent with our original phenotypic data (Figure 2, B and C).

We constructed a heatmap to visualize the transcriptional expression of the markers used in our flow cytometry panel in Figure 1 and observed that the expression patterns in our transcriptional data set were consistent with phenotypic data (Figure 2, D and E). Pregnancy induced comparable levels of *Pdcd1* and higher levels of exhaustion-associated transcripts *Lag3* and *Tigit* in R+P C5 and C7 compared with P C5. In contrast, P C5 expressed higher levels of the anergy-associated transcripts, Nt5e (CD73) and Izumo1r (FR4) compared with R+P C7.

We next performed differential expression analysis to visualize the global transcriptional differences via UMAP (Figure 2F). R and N T_FGS_ displayed distinct transcriptional signatures, with the P C1 subset nearly identical to N T_FGS_ and the R+P C4 subset similar to R T_FGS_. These data corroborate the phenotyping data (Figure 1, H–J) that a subset of T_FGS_ remained unmodified by pregnancy in both P and R+P mice. Differentially expressed genes (DEGs) by C5 T_FGS_ from P and R+P indicated that they were transcriptionally similar, while R+P C7 vs. R+P C5 T_FGS_ were more similar than initially anticipated based on the phenotypic data, sharing ~60% of their transcriptome respectively (Figure 2G).

**Figure 3:**
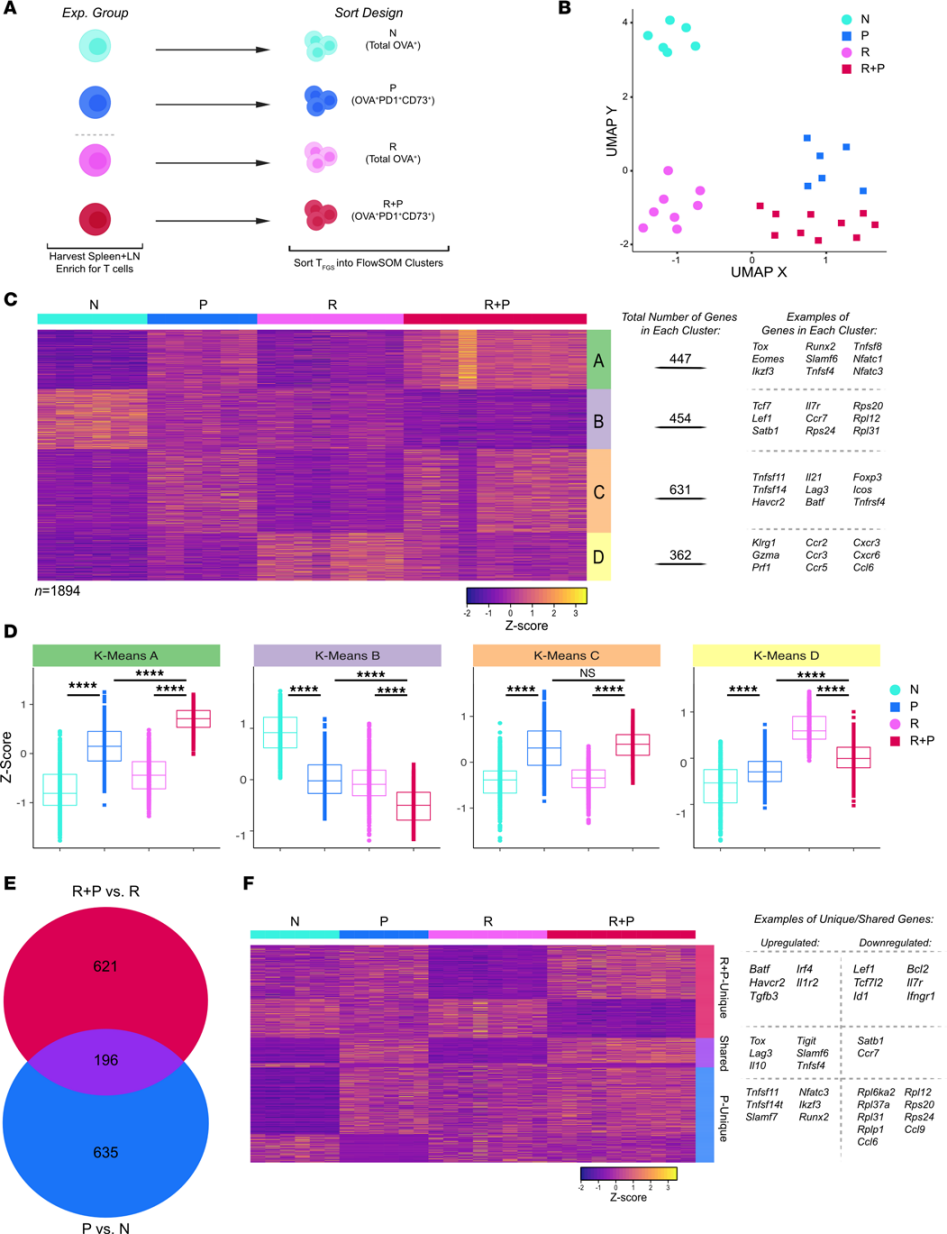
Postpartum memory and naive OVA-specific T_FGS_ acquire distinct and shared transcriptional signatures. (**A**) OVA:K^b^-specific T_FGS_ from each experimental group; N, R, P (C5), and R+P (C5). (**B**) UMAP plot comparing transcriptional profiles among T_FGS_ subsets. (**C**) Row-normalized RNA-Seq expression of the top differentially expressed genes (*n* = 1,894), organized by K-means clustering into Clusters A–D, indicated by right-side column. The total number and examples of DEGs in each K-means cluster are listed on the right. (**D**) Box plots visualizing relative expression of DEGs in each K-Means cluster identified in **C**. Minimum criteria for DEGs shown in this figure was both *q* < 0.1 and log_2_ fold-change > 0.9. *P* values (in **D**) were determined by Kruskal-Wallis 1-way ANOVA with Dunn’s post hoc test. ****P < 0.0001. (**E** and **F**) Venn diagram and row-normalized RNA-Seq expression of DEGs induced by pregnancy in only R+P (*n* = 635 DEGs), only P (*n* = 621 DEGs), or both R+P and P T_FGS_ (*n* = 196 DEGs). Each dot in UMAP or box plots, and each column in the heatmap, indicates an individual mouse.

To reduce complexity, we henceforward focused our transcriptional analysis on the postpregnancy cell clusters P C5 and R+P C5, referring to them P and R+P, respectively (Figure 3A). UMAP confirmed that the transcriptomes of these postpartum P and R+P cells were more similar to each other than before pregnancy (Figure 3B). Visualizing the top DEGs between the 4 experimental groups (N, P, R, R+P) by heatmap and K-means clustering identified 4 main DEG clusters (Figure 3, C and D). Clusters A and C DEG were upregulated by pregnancy in P and R+P compared with N and R T_FGS_–included T cell exhaustion (T_EX_) genes, *Tox*, *Eomes*, *Slamf6*, *Nfatc1/3*, *Lag3*, and *Havcr2* (Tim-3). Cluster B DEGs were downregulated in R and P — and even more so in R+P — included *Tcf7* and *Lef1* transcriptional factors that are reduced in T_EX_ (29–31). Interestingly, DEG Cluster D (*n* = 362) was strongly upregulated in R vs. N but downregulated in R+P T_FGS_ to levels that approached P T_FGS_. Metascape analysis categorized these DEGs as enriched for negative regulation of inflammatory responses, NK cytotoxicity, lymphocyte immunity, and viral protein interaction genes (Supplemental Figure 2, A–E). These DEGs included critical T cell effector genes (*Gzma*, *Prf1*) as well as chemokine genes that control effector T cell migration to tissue sites (*Cxcr3*, *Ccr5*, *Ccr6*, *Ccr2*) (32–35). These data raise the possibility that a subset of memory-associated upregulated transcripts is significantly downregulated by pregnancy.

We next focused our analysis on the unique 817 and 831 DEGs induced by pregnancy, with a 24% (*n* = 196) transcriptional overlap in R+P and P T_FGS_, respectively (Figure 3E). Visualizing DEGs unique to postpartum memory T_FGS_ via heatmap and volcano plots showed comparable numbers of upregulated genes (including exhaustion-associated genes: *Ikzf2* [HELIOS], *Havcr2* [TIM-3]), and downregulated genes (including memory-associated genes: *Lef1*, *Il7r* [CD127], and *Tcf7l2* [TCF-4]) (Figure 3F and Supplemental Figure 3A) (29–31, 36, 37). Metascape pathway analysis of R+P-unique DEGs (vs. R) indicated an upregulation of the regulatory pathways for cytokine production and T cell differentiation as well as downregulation of JAK-STAT and Delta-Notch signaling (Supplemental Figure 3B). In contrast, the majority of DEGs unique to P T_FGS_ were upregulated, including *Nfatc3*, *Ikzf3*, and *Runx2*, and were within the T cell costimulation and cellular response to IL-18 Metascape pathways (Figure 3F and Supplemental Figure 3C).

Finally, we examined the set of 196 shared DEGs induced by pregnancy in both memory and N T_FGS_, with the majority of these DEGs being upregulated (168 genes) (Figure 3F). Metascape pathway analysis revealed an enrichment in regulation of cytokine production and of T cell activation and differentiation (Supplemental Figure 3D). Notable examples included upregulated *Tox*, *Nfatc1*, *Il10*, *Il21,* and *Tnfsf4* and downregulated *Ccr7* and *Satb1* (Supplemental Figure 3E). In contrast, R T_FGS_ displayed distinct transcriptional signatures, with many of the upregulated genes classified in T cell activation and effector function pathways (Supplemental Figure 4, A and B). Taken together, these data show that the induction of T cell hypofunction by pregnancy results in shared and distinct transcriptional changes in memory vs. N T_FGS_.

### Pregnancy elicits an exhausted transcriptional signature in memory T_FGS_.

**Figure 4:**
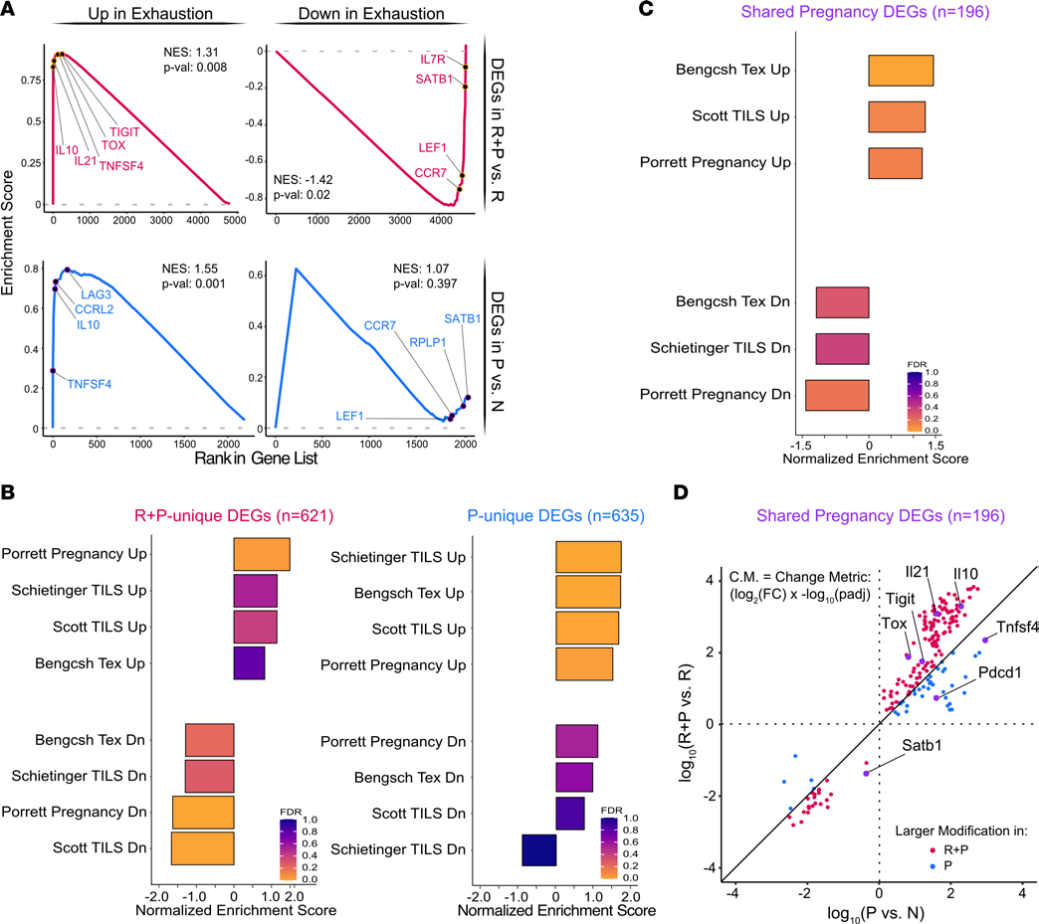
Postpartum memory OVA-specific T_FGS_ acquire a transcriptional signature of exhaustion. (**A**) GSEA curves showing enrichment of the exhausted T cell signature (chronic viral infection; ref. 38) in R+P vs. R and P vs. N DEGs. NES, normalized enrichment score. (**B** and **C**) Summary of GSEA comparing DEGs unique to R+P vs. R (left) or P vs. N (right) (**B**), and shared DEGs by R+P and P to published gene sets of exhaustion (**C**) (6, 38–40). (**D**) Dot plot comparing magnitude of up- or downregulation for shared DEGs between R+P and P T_FGS_ using the Change Metric (C.M.), a single statistic that merges FDR-corrected *P* value and log fold change (±log_2_[FC] × –log[FDR]). *P* values were calculated with the Wilcoxon matched-pairs signed rank test comparing R+P vs. P T_FGS_. P(upregulation) < 0.0001; P(downregulation) = 0.0032.

Lewis et al. (6) recently reported that pregnancy-induced hypofunction in N OT-I cells was associated with a transcriptional state of exhaustion, prompting us to test whether memory T_FGS_ could be similarly reprogrammed into exhaustion. To this end, we ranked the DEGs induced by pregnancy in memory or N T_FGS_, comparing them to hallmark gene sets of T_EX_ during chronic infection by Gene Set Enrichment Analysis (GSEA) (Figure 4A) (38). Indeed, we observed a significant enrichment in both upregulated and downregulated T_EX_ signatures in R+P T_FGS_. Notably, *Tox* and *Tigit* were identified as part of the leading edge of upregulated genes, while *Satb1* and *IL-7r* were in the leading edge of downregulated genes. In contrast, GSEA of the DEGs induced in P T_FGS_ revealed a significant enrichment of only the upregulated T_EX_ signature. As a control, we also ran GSEA on the DEGs of rejection (R vs. N T_FGS_) and showed that there was no enrichment for any exhaustion gene sets (Supplemental Figure 4C). Taken together, these GSEA supported a more enriched transcriptional response toward exhaustion in postpartum memory compared with N T_FGS_, consistent with the hypothesis that more coinhibition is required to constrain memory T_FGS_.

We performed GSEA on the distinct and shared DEGs induced by pregnancy in N and memory T_FGS_, and we compared them with multiple additional T_EX_ gene sets from cancer, chronic infection, and pregnancy (Figure 4, B and C, and Supplemental Figure 5) (38–40). We corroborated the observation that pregnancy-induced DEGs unique to R+P were enriched for both up- and downregulated T_EX_ genes, whereas only upregulated T_EX_ transcripts were enriched in P. Furthermore, even within the 196 pregnancy-induced gene set shared by N and memory T_FGS_, we observed a statistically significant trend that the relative magnitude of transcriptional change was greater in R+P vs. P T_FGS_ (Figure 4D). Notable examples of DEGs following this trend included *Tox*, *Tigit*, *Il10*, *Il21*, and *Satb1*. In contrast, only a small subset of genes was more upregulated in P, including *Pdcd1* (PD-1) and *Tnfsf4* (OX40L).

Taken together, our RNA-Seq data confirm that pregnancy induces clearly distinct global transcription signatures in memory vs. N T_FGS_, with significantly higher levels and more extensive expression of exhaustion-associated transcripts in memory T_FGS_.

### Pregnancy induces distinct phenotypes of hypofunction in memory vs. N T_FGS_.

**Figure 5:**
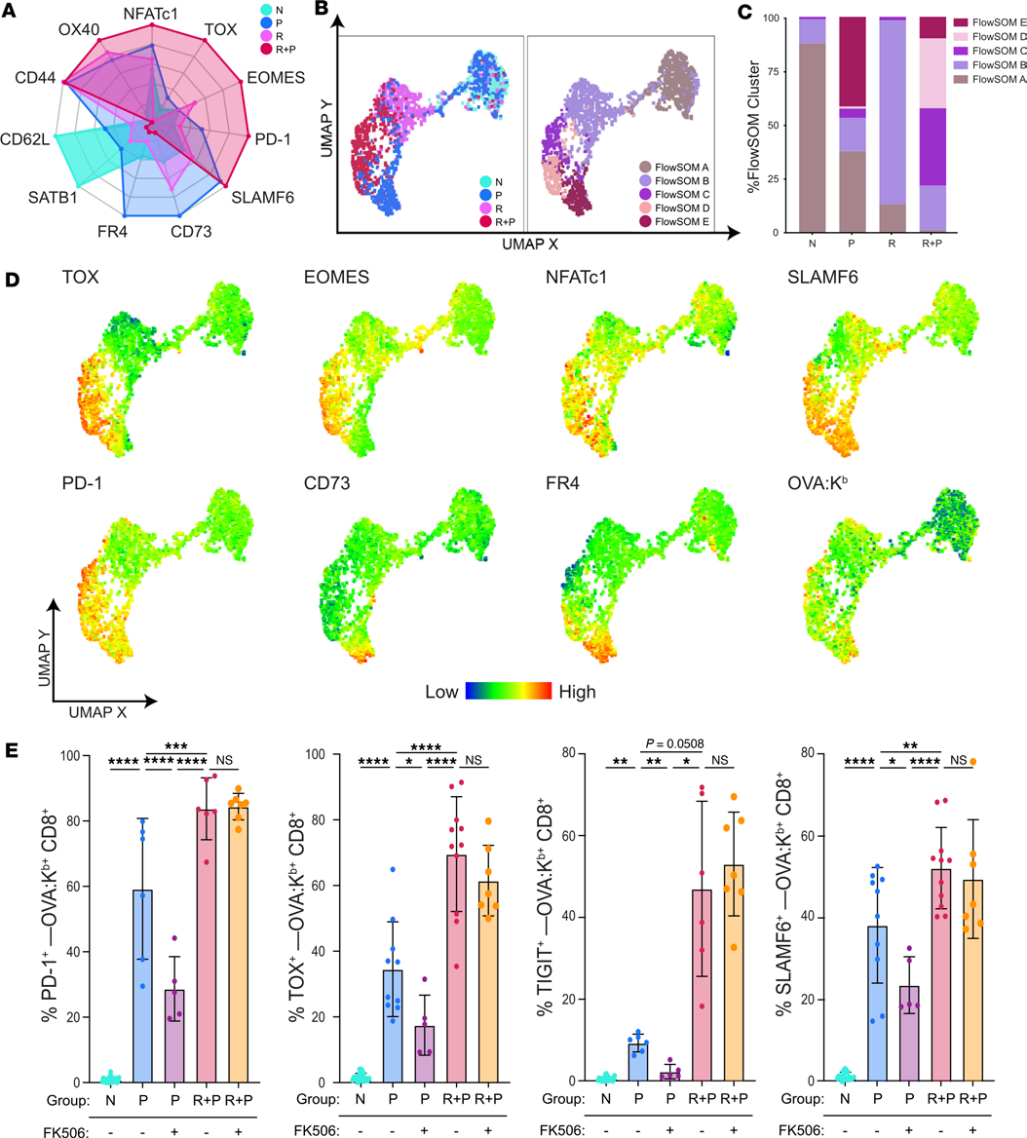
Pregnancy programs distinct exhaustion phenotypes in memory vs. naive OVA-specific T_FGS_. (**A–E**) Flow cytometry panel based on RNA-Seq results confirms phenotypic exhaustion in postpartum T_FGS_. (**A**) Radar plot presenting normalized expression of phenotypic markers (based on highest and lowest MFI for each marker expressed by T_FGS_ and non-T_FGS_ from all 4 experimental groups) demonstrates enhanced separation between R+P and P T_FGS_. (**B** and **C**) UMAP and FlowSOM reveal distinct clusters for R+P and P T_FGS_ driven by phenotypic differences in TOX, EOMES, FR4, and CD73. (**D**) UMAP with heatmap overlays were generated to visualize phenotypic differences between T_FGS_ subsets. (**E**) Expression levels of PD-1, TOX, TIGIT, and SLAMF6 by memory vs. naive T_FGS_ from dams treated with FK506, an inhibitor of NFAT. *P* values were determined by 1-way ANOVA; *P < 0.05; **P < 0.01; ***P < 0.001; ****P < 0.0001.

We validated our transcriptional findings by developing a larger 23-color spectral flow cytometry panel to assess the phenotypic expression of additional markers identified in our transcriptional analysis (Supplemental Table 1). This panel more readily captured differences between P and R+P T_FGS_, as illustrated by radar plot and UMAP + FlowSOM (Figure 5, A–C), that were not observed in the non–OVA-specific CD8^+^ T cells (Supplemental Figure 6A). P T_FGS_ (Cluster E) preferentially upregulated the anergy markers, FR4 and CD73, and more modestly upregulated T_EX_ markers, Tox and Eomes, compared with R+P T_FGS_ (Figure 5, B–D, and Supplemental Figure 6, B–G). The majority of R+P T_FGS_ mapped to Clusters C and D, which were characterized by a significantly more robust expression of Tox and Eomes compared with P T_FGS_. These data highlight the distinct gradation of phenotypic exhaustion markers induced by pregnancy in memory vs. N CD8^+^ T_FGS_.

Observations by Lewis et al. (6) and our observation of induced expression of NFAT in memory vs. N T_FGS_ prompted us to test whether the phenotypic profiles of exhaustion were dependent on NFAT signaling. We show that treatment with FK506, a pharmacological inhibitor of NFAT, during pregnancy significantly reduced the expression of exhaustion markers PD-1, Tox, NFATc1, Tigit, and SLAMF6 and the anergy marker CD73 in P T_FGS_ consistent with Lewis et al. (6) (Figure 5E and Supplemental Figure 7, A–C). Notably, these markers were not significantly inhibited in R+P T_FGS_, suggesting that the expression of exhaustion/anergy markers in P T_FGS_ is partially dependent on NFAT signaling, whereas their expression by R+P T_FGS_ is NFAT independent. This underscores another difference in how pregnancy affects N vs. memory T_FGS_ and raises the possibility of differential epigenetic modification driving the T_EX_ phenotype in R+P T_FGS_.

### Pregnancy programs extensive exhaustion-associated chromatin remodeling in memory T_FGS_ but not in N T_FGS_.

Because CD8^+^ T cells undergo epigenetic modifications during the differentiation into effector/memory and exhausted/hypofunctional T cells (37, 41–45), we hypothesized that pregnancy would epigenetically program memory and N T_FGS_ to sustain their states of hypofunction. We used the same sorting strategy described for RNA-Seq as defined in Figure 2 on T_FGS_ to perform the assay for transposase-accessible chromatin with high-throughput sequencing (ATAC-Seq). Chromatin accessibility heatmaps provided a visualization of global differences between T_FGS_ subsets, while pie charts show comparable genomic distribution of the reproducible ATAC-Seq peaks identified for each T_FGS_ cluster (Supplemental Figure 8, A and B). An UpSet plot showed the total number of reproducible peaks shared by various combinations of T_FGS_ subsets, noting unique peaks present only in R+P C5 and/or C7 T_FGS_ (Supplemental Figure 8C).

By visualizing the differentially accessible peaks (DAPs) using UMAP and heatmap with K-means clustering, we show that N vs. R T_FGS_ had distinct chromatin accessibility profiles (K-means clusters A and B) consistent with the acquisition of a memory T cell epigenome (Supplemental Figure 9, A–D). These clusters grouped loci that were remodeled in R and more extensively in postpartum memory (R+P) T_FGS_. In contrast, K-means cluster C grouped loci that were closed in N, P, R, and the R+P C4 (PD-1^neg^) subsets but significantly opened in R+P C5 and C7 T_FGS_. Finally, K-means cluster D loci were open in N, P, and R subsets but closed in R+P T_FGS_ (46–49). Collectively, these observations support the hypothesis that pregnancy imposed more extensive epigenetic modulation in memory vs. N T_FGS_.

**Figure 6:**
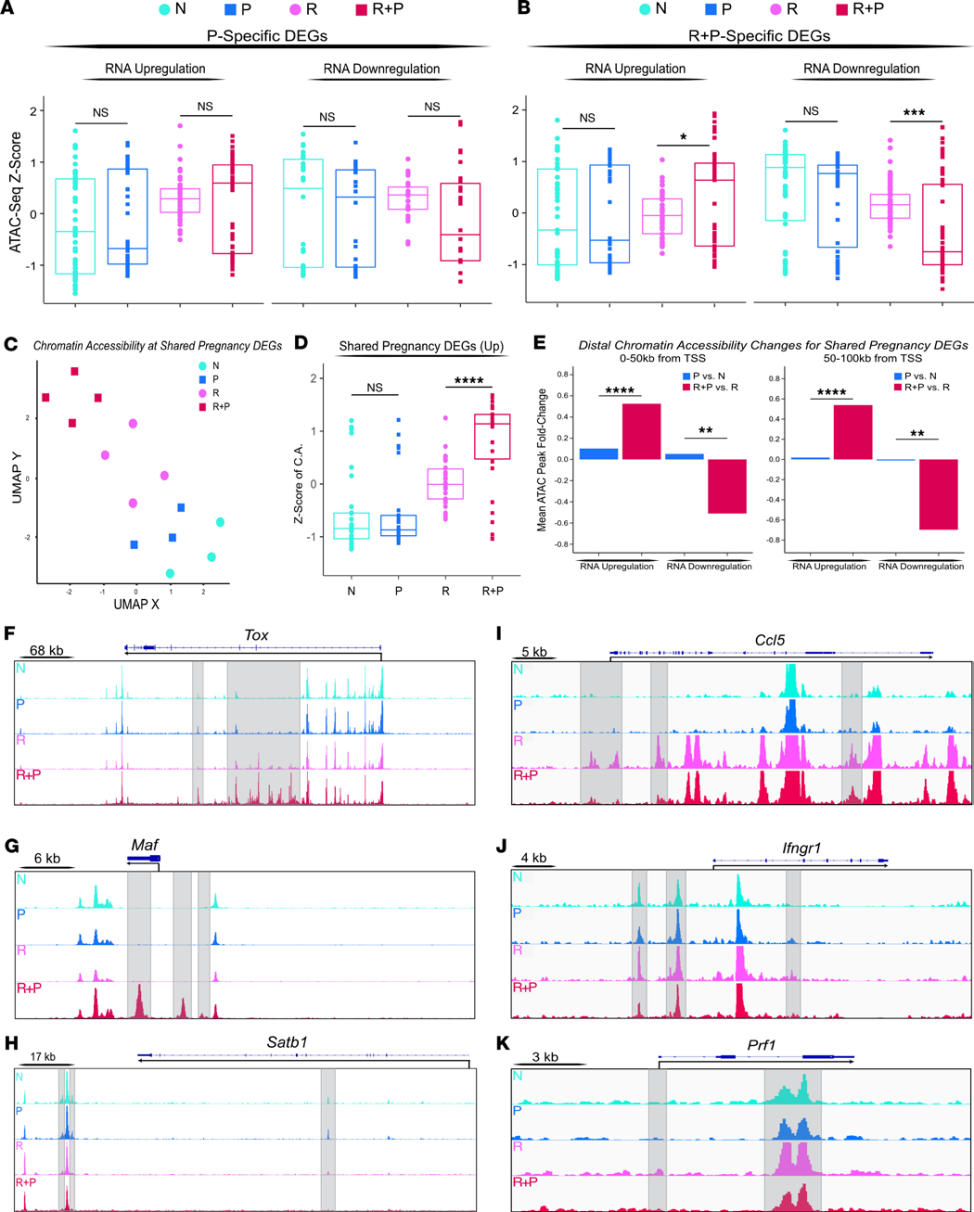
Pregnancy alters the chromatin state of memory but not naive OVA-specific T_FGS_. (**A** and **B**) T_FGS_ subsets were acquired and sorted for ATAC-Seq as in Figure 2A. Box plots visualizing chromatin accessibility at DEGs unique to P vs. N (**A**) or unique to R+P vs. R (**B**). *P* values (for **A** and **B**) were determined by Welch’s 2-tailed *t* test. (**C** and **D**) UMAP and box plots of chromatin accessibility at the 196 DEGs shared by P and R+P vs. R T_FGS_. Data acquired from ≥ 2 biologically independent experiments with *n* = 3–4 per group. *P* values (for **D**) were determined by Welch’s 2-tailed *t* test. (**E**) Bar plots visualizing the mean fold-change of distal ATAC-Seq peaks within 0–50 kb (left) or 50–100 kb (right) of the TSS of shared pregnancy-induced DEGs. P vs. N T_FGS_ (blue) or R+P vs. R T_FGS_ (red). *P* values (for **E**) were determined by Welch’s 1-tailed *t* test. (**F–K**) ATAC-Seq tracks at the *Tox*, *Maf*, and *Satb1* loci (**F–H**), and *Ccl5,* *Ifngr1*, and *Prf1* (**I**–**K**). Peaks uniquely induced in R and reversed in R+P T_FGS_ are highlighted in gray. Each dot in box plots or UMAP indicates an individual mouse. **P < 0.01, ***P < 0.001; ****P < 0.0001.

To more rigorously address the hypothesis that epigenetic modifications in R+P but not P T_FGS_ occurred during pregnancy, we leveraged our RNA-Seq data set from Figure 3C to assess chromatin remodeling associated with pregnancy-induced DEGs in P or R+P T_FGS_. At the loci of all DEGs (*n* = 831) uniquely induced in N T_FGS_ by pregnancy, we observed no significant change in chromatin accessibility (Figure 6A). In contrast, significant increases and decreases in chromatin accessibility in the DEGs (*n* = 817) induced by pregnancy in memory T_FGS_, corresponding to transcriptional up- and downregulation, respectively (Figure 6B). These observations support the hypothesis that exhaustion transcriptome was associated with extensive pregnancy-mediated chromatin remodeling uniquely in memory T_FGS_, while the exhaustion transcriptome in N T_FGS_ required minimal chromatin remodeling.

Supporting this conclusion, chromatin accessibility of the 196 shared DEGs induced by pregnancy and enriched for T_EX_ in R+P and P T_FGS_ was also significantly changed in R+P vs. R T_FGS_ but not in P vs. N T_FGS_ (Figure 6, C and D). Notably, pregnancy-mediated chromatin remodeling remained detectable at distances of up to 100 kb from the transcription start sites of these loci, supporting the possibility of both proximal remodeling of the locus itself and distal enhancer remodeling (Figure 6E). These differences are readily apparent when visualizing individual exhaustion-associated loci such as *Tox* and *Maf*, where multiple open peaks were present in R+P T_FGS_ but not in R or P T_FGS_ (Figure 6, F and G) (36, 37, 40, 50, 51). Chromatin accessibility of *Satb1* was reduced in R+P, consistent with reduced transcription and its ability to repress PD-1 expression in CD8^+^ T cells (Figure 6H) (52). Notably, increased chromatin accessibility in R and decreased in R+P T_FGS_ were also observed for T cell effector/memory genes *Prf1*, *Ccl5*, *Ifngr1*, *FasL,* and *Gata3,* which were transcriptionally downregulated (Cluster D, Figure 3D) in postpartum memory T_FGS_ (Figure 6, I–K, and Supplemental Figure 10, A and B) (29, 30, 48, 53).

Finally, HOMER de novo motif analysis was used to search for enrichment of conserved transcription factor DNA binding motifs associated with T cell function and differentiation among the DAPs in R+P vs. R T_FGS_ (Supplemental Figure 11). This analysis identified, in R+P vs. R T_FGS_, key motifs closing for *Lef1*, *Tcf7,* *Tcf4,* *Tcfl2*, *Batf* and motifs opening for *Nfatc1*, *Tbx21*, *Eomes*, *Runx*, and *Jun* that have been implicated in T cell exhaustion (48). Together, these data support the conclusion of extensive epigenetic modification in postpartum memory T_FGS_ at loci involved in T cell exhaustion and in a subset of the memory T cell signature, and that involve key transcription factor binding.

### Pregnancy programs sustained hypofunction in memory T_FGS_.

**Figure 7:**
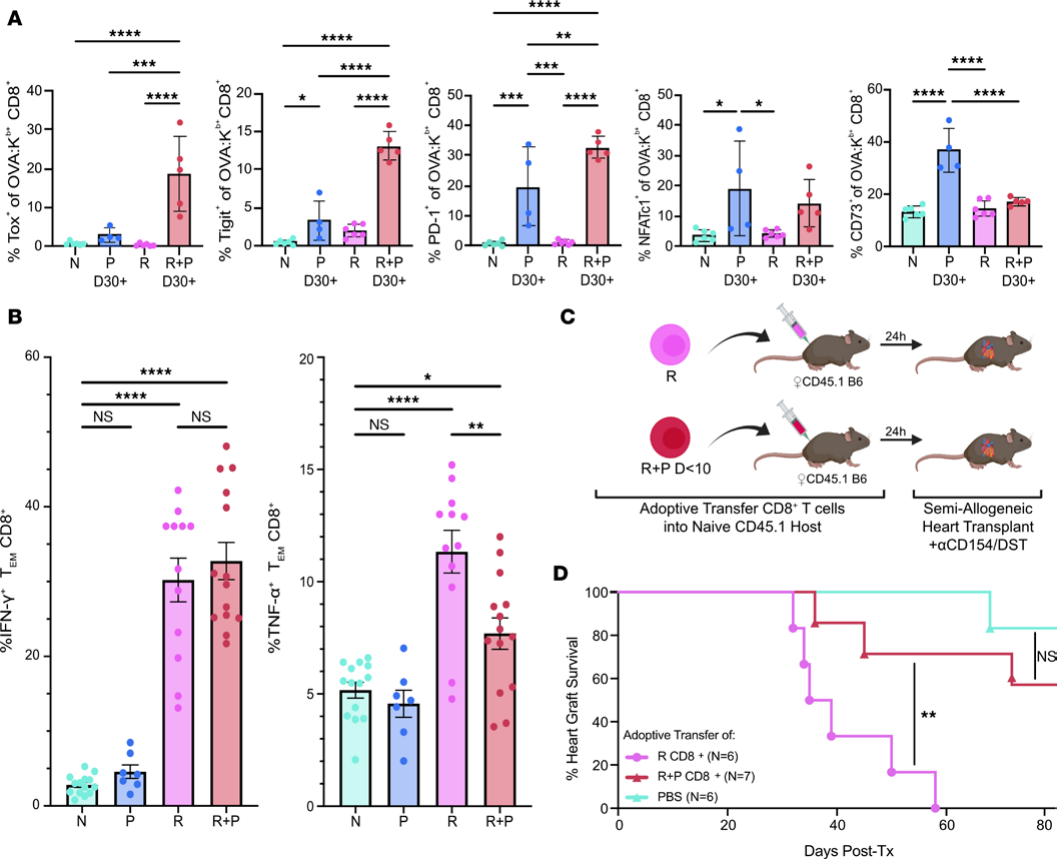
Pregnancy induces in memory OVA-specific T_FGS_ a sustained exhausted phenotype and restores susceptibility to costimulation blockade–induced acceptance of fetus-matched heart allografts. (**A**) Percentage of OVA-specific T_FGS_ from P and R+P (both at postpartum day 30), Naive (N) or R (days 30–60 after skin transplant) expressing Tox, Tigit, PD-1, NFATc1, and CD73. (**B**) Bar graphs visualizing IFN-γ (left) and TNF-α (right) production of CD8^+^ T_EM_ cells (CD44^hi^CD62L^–^) after overnight in vitro stimulation with activated F1 APCs. Data were acquired from 2 or more biologically independent experiments; *n* = 4–13 per group. Data represent mean ± SEM. *P* values were determined by 1-way ANOVA (**A**) and Kruskal-Wallis 1-way ANOVA test with Dunn’s post hoc test (**B**). (**C**) Experimental design for adoptive transfer (AdTr) of CD8^+^ T cells from R or R+P mice (harvested on postpartum days 0–10) into naive CD45.1 B6 mice. One day after AdTr, these and PBS-control mice received allogeneic 2W-OVA.F1 (2W-OVA.B/c × B6) heart transplantation with anti-CD154/DST tolerance induction. (**D**) Percentage of 2W-OVA.F1 heart graft survival among AdTr recipients; *n* = 6–7 per group. Each dot indicates an individual m ouse. *P* values were determined by Mantel-Cox log-rank test. *P < 0.05; **P < 0.01, ***P < 0.001; ****P < 0.0001.

Because T cell exhaustion is diminished upon antigen deprivation, we tested whether the exhaustion phenotype induced by pregnancy was persistent in P and R+P T_FGS_ (45, 54). On post–skin transplant (days 30–60) or postpartum days 30–37, the expression of CD44 was significantly increased, and CD62L was significantly reduced (Supplemental Figure 12, A and B). The levels of exhaustion markers Tox, Tigit, and PD-1 by R+P T_FGS_ remained significantly elevated compared with R or P T_FGS_ (Figure 7A). In contrast, the expression of NFAT and FR4 was comparable in R+P and P T_FGS_, while CD73 was highest in P T_FGS_ (Figure 7A and Supplemental Figure 12C). These data suggest that pregnancy-induced exhaustion was persistent, especially in postpartum memory compared with N T_FGS_.

We next quantified the in vitro cytokine production capability of CD8^+^ T cells following stimulation with allogeneic APCs. As expected, ~12% and 30% of R T_FGS_ produced TNF-α and IFN-γ, respectively, which is significantly higher than N T_FGS_ (Figure 7B and Supplemental Figure 13). P T_FGS_ exhibited minimal TNF-α and IFN-γ production, remaining comparable with N T_FGS_. Notably, TNF-α production was significantly reduced in R+P T_FGS_ compared with R T_FGS_; however, the ability to IFN-γ was not significantly altered (Figure 7B).

Finally, we tested whether the recall encounter of fetal antigens by memory T cells during pregnancy resulted in a persistent hyporesponsive state in the context of offspring-matched heart transplantation. To avoid the humoral sensitization that is simultaneously elicited by pregnancy and that we have previously shown as sufficient to mediate rejection of F1 heart grafts (3), we used an adoptive transfer (AdTr) approach whereby CD8^+^ T cells purified from R or R+P (postpartum days 0–10) mice were injected into N B6 hosts. Following AdTr of CD8^+^ T cells, B6 hosts were transplanted with an F1 heart graft (B6 × BALB/c) and received anti-CD154 and donor splenocyte transfusion (DST; anti-CD154/DST) (Figure 1, D and E), a therapy that induces long-term graft acceptance in N hosts. Consistent with previous reports (17, 18), memory CD8^+^ T cells from R mice prevented stable graft acceptance. Remarkably, anti-CD154/DST treatment induced a significant extension of allograft survival in recipients of R+P CD8^+^ T cells (Figure 7, C and D, and Supplemental Table 2). Thus, pregnancy enforces a cell-intrinsic state of hypofunction in postpartum memory T_FGS_ that manifests as restored susceptibility to anti-CD154/DST–induced tolerance of offspring-matched heart grafts.

## Discussion

Most studies of T cell tolerance to the semiallogeneic fetus investigated the immunological effects of pregnancy in N mice or those sensitized by prior pregnancy; in contrast, we show that the processes evoked during pregnancy are capable of restraining alloreactive memory T and B cell responses generated by skin graft rejection to allow for full-term delivery of viable semiallogeneic offspring. The potential mechanisms mediating the reprogramming of memory CD8^+^ T cells to hypofunction by pregnancy are suggested by their phenotypic and transcriptional signatures, which illustrated the differential effect pregnancy had on memory vs. N T_FGS_. Postpartum memory T_FGS_ had significantly higher transcriptional and phenotypic expression of exhaustion markers Tox, Eomes, PD-1, and Tigit, whereas postpartum N T_FGS_ preferentially expressed the anergy markers FR4 and CD73. GSEA confirmed that pregnancy-induced transcripts in R+P were significantly enriched for canonical CD8^+^ T cell exhaustion signatures that were up- and downregulated in CD8^+^ T cells infiltrating tumors or in chronic infection. In contrast, P T_FGS_ were enriched for only upregulated transcripts associated with exhaustion. Additionally, even within the shared 196 DEGs induced by pregnancy in both N and memory T_FGS_, the magnitude of up- or downregulation was significantly greater in R+P compared with P T_FGS_. We hypothesize that the higher levels of exhaustion and coinhibitory markers are required to successfully restrain memory T cells, which have lower levels of activation thresholds due to increased TCR avidity and epigenetic programming (55, 56).

Changes in chromatin accessibility are the result of histone methylation, acetylation, and phosphorylation that allow for increased or reduced transcriptional factor binding and subsequent gene transcription (57). We observed that pregnancy uniquely induced chromatin remodeling in memory CD8^+^ T_FGS_, whereas N T_FGS_ remained largely epigenetically unmodified by pregnancy, even at shared exhaustion-associated loci induced transcriptionally by pregnancy. Additionally, pregnancy-induced opening of chromatin in postpartum memory T_FGS_ was enriched for transcription factor motifs implicated in both early- and late-stage T cell exhaustion, including *Tbx21*, *Eomes*, and *Jun* (48). These observations are congruent with the significant increase in transcription of exhaustion-associated genes in postpartum memory T_FGS_. The idea that epigenetic modification enforces the hypofunctional state may provide an explanation for the resistance to NFAT inhibition seen in R+P compared with P T_FGS_. The basis for why pregnancy has distinct chromatin remodeling effects in R+P vs. P T_FGS_ is unclear, but we speculate that it may be due to intrinsically distinct epigenetic landscape in N vs. R T_FGS_ from which R+P and P T_FGS_ are derived.

Our observations also support the hypothesis that the partial reduction of the memory transcriptome and epigenome contributes to the hypofunctional state of R+P T_FGS_. In addition to the upregulation of DEGs that negatively regulate cytokine production (*Havcr2*, *Pdcd1lg2*, *Tgfb3*), multiple genes involved in the rapid response upon antigen reencounter were downregulated in R+P vs. R T_FGS_. These include genes encoding effector molecules (*CD48*, *Prf1*, *Fasl*, *Fcgr2b*, *Klr* family), chemokine/chemokine receptors (*Ccl6*, *Ccl9*, *Ccl5*, *Ccr2*, *Ccr3*, *Cxcr3*, *Cx3cr1*, *Xcr1*), and cytokine/ cytokine receptors (*Il18r1*, *Il18r1*, *Ifngr1*, *Il2rb*) (34, 45, 53, 55). Furthermore, a subset of these genes (*Ccl5*, *Gata3*, *Ifnr1*, *Prf1*, and *Fasl*) that underwent chromatin remodeling following rejection was reversed by pregnancy. These data raise the possibility that pregnancy utilizes targeted epigenetic modifications in memory T_FGS_ not only to induce transcriptional exhaustion but also to dedifferentiate T_FGS_ from memory/effector programs.

Memory CD8^+^ T_FGS_ generated following rejection of allogeneic skin grafts exhibit increased production of TNF-α and IFN-γ and resistance to costimulation blockade–mediated acceptance of heart allografts compared with N T_FGS_ (3, 16–18). Postpartum memory CD8^+^ T cells exhibited significantly reduced ability to produce TNF-α, but retained their ability to produce IFN-γ, relative to R CD8^+^ T cells. The physiological roles of uterine NK cells producing IFN-γ in promoting pregnancy, remodeling vascular/tissue, and preventing excessive trophoblast invasion have been described (58, 59). Furthermore, TNF-α combined with high doses of IFN-γ is compatible with healthy pregnancy, and “controlled” levels of Th1 cells and TNF-α may have essential roles in successfully pregnancy (58, 60, 61). Thus, we speculate that the ability to produce IFN-γ and TNF-α by T cells may be preserved in pregnancy. It is notable that IFN-γ plays a nonredundant role in allograft tolerance, as mice deficient in IFN-γ fail to develop tolerance with no defects in acute rejection (62, 63). Indeed, we show that pregnancy was able to relieve the barrier memory CD8^+^ T cells normally pose to transplantation tolerance, as evidenced by the enhanced survival of subsequent offspring-matched heart grafts under costimulation blockade in recipients that received R+P vs. R CD8^+^ T_FGS_. These observations provide proof-of-concept that memory CD8^+^ T cells, which heretofore were considered an insurmountable barrier to clinical transplantation tolerance, can be reprogrammed to hypofunction and susceptibility to anti-CD154/DST–induced graft acceptance.

There are several limitations to this study. Firstly, we introduced a model OVA antigen to the semiallogeneic fetus and allograft to enable tracking endogenous polyclonal fetus/graft specific CD8^+^ T cells. It is possible that the highly immunogenic OVA may be immunodominant over “true” alloantigens and elicit higher avidity T cell responses than observed for alloreactive T cells. Secondly, our data correlate the expression of exhaustion transcriptome and markers, as well as the partial reversal of the memory phenotype, with the hypofunctional state of postpartum memory T_FGS_. However, definitive necessity and sufficiency studies are necessary. Thirdly, a mechanistic explanation for why memory but not N T_FGS_ undergo such extensive chromatin remodeling during pregnancy is lacking, and necessity of this remodeling for the maintenance of the hypofunction state in postpartum memory T_FGS_ has not be demonstrated. Finally, while sensitivity to costimulation blockade is significantly restored, all the F1 heart grafts ultimately rejected. Postpartum memory T_FGS_ retained the ability to produce IFN-γ and TNF-α, and a subset of R T_FGS_ were unmodified by pregnancy. These observations suggest that more extensive and additional memory programs may have to be constrained to achieve comparable states of hypofunction as observed in postpartum N T_FGS_ (3).

Pregnancy is an immunological paradox, wherein the conflict between the preservation of robust immunity toward foreign pathogens and tolerance to the semiallogeneic fetus must be simultaneously resolved to preserve the survival of the species. Furthermore, memory fetus–specific T cells must be constrained. The imperative to preserve fetal viability underscores the necessity of multiple conserved and redundant mechanisms for controlling both naive and memory T cells. Our studies reveal a potentially novel endogenous mechanism for the reprogramming of antigen-specific memory T cells toward exhaustion and hypofunction (Supplemental Figure 14). This mechanistic insight is critically relevant for understanding semiallogeneic pregnancy as well as the successful induction of transplantation tolerance in the clinic, where no conceptual framework for reprogramming of memory donor-specific T cells has yet been identified (16–18). In addition, viewing CD8^+^ T cell exhaustion/hypofunction through the lens of pregnancy potentially solves the seemingly counterintuitive evolutionary puzzle of why exhaustion is so quickly induced when T cells are exposed to chronic infections or tumors, which is often detrimental to the host (64). We theorize that this timeline is imposed by mammalian pregnancy requiring a rapid restraint of fetus-specific alloreactive T cells to preserve fetal viability. Moreover, while the phenotype and transcriptome of exhaustion was initially discovered in the context of chronic infection and tumors, we posit that this phenomenon should be reevaluated from the perspective that exhaustion pathways developed due to the stringent need to preserve the semiallogeneic fetus, and these mechanisms have been subsequently hijacked by chronic infections and tumors. Thus, insights into how exhaustion is programmed into memory T_FGS_ during pregnancy are relevant not only to addressing problems related to high-risk pregnancies and transplantation tolerance but also to broader clinical issues such as autoimmunity, chronic infection, and cancer, where controlling T cell hypofunction is also desirable.

## Methods

### Sex as a biological variable.

This study’s main focus is the effect of pregnancy on the maternal immune system. The investigation of pregnancy justifies and necessitates the use of exclusively female mice in this study.

### Mice.

Eight- to 12-week-old female B6 (H-2^b^) mice were purchased from Harlan Laboratories. *Act-2W-OVA* transgenic mice on a B/6 background (2W-OVA.C57BL/6) were a gift from James Moon (Massachusetts General Hospital, Harvard Medical School, Charlestown, Massachusetts, USA). Donor/paternal 2W-OVA.BALB/c (2W-OVA.B/c, H-2^d^) mice were backcrossed from 2W-OVA.B/6 mice for > 10 generations. For semiallogeneic pregnancies, harem breeding involved 1 male 2W-OVA.BALB/c with 3–4 N or R B/6 females. Approximately 50% of F1 from this mating were confirmed to be 2W-OVA^+^, and 2W-OVA.F1 (B6 × 2W-OVA.B/c) mice were used as heart donors.

### AdTr, heart, and skin transplantation.

For AdTr experiments, ~4 12 × 10^6^ to 12 × 10^6^ CD45.2^+^ CD8^+^ T cells, isolated via magnetic enrichment, were transferred retroorbitally (r.o.) into N CD45.1^+^ B6 hosts 1 day prior to heart transplantation. See below for T cell enrichment description. Heterotopic heart transplantations were performed as previously described (65), by grafting 2W-OVA.F1 (B6 × 2W-OVA.B/c) hearts onto the inferior vena cava and aorta of female recipients. Tolerance (CoB/DST) was induced with a combination of anti-CD154 (MR1, BioXCell) at a dose of 500 μg on day 0 (i.v.), and 250 μg on days 7 and 14 (i.p.) posttransplantation, in combination with 2 × 10^7^ donor spleen cells on day 0. Graft survival was assessed by palpation 2–3 times per week, and the day of rejection was defined as the last day of detectable heartbeat. Flank skin from 2W-OVA.BALB/c was transplanted onto B/6 mice.

### FK506 injection.

FK506 was injected daily (1 mg/kg i.p.) into pregnant mice beginning 5 days after the first observation of a copulation plug and ending on the date of euthanasia (days 0–3 after delivery).

### T cell enrichment.

Single-cell suspensions from spleens and pooled lymph nodes (LNs) (brachial, inguinal, and axillary) of individual mice were prepared for each experiment (see below). For flow cytometry and cell sorting assays, Pan-T lymphocytes were enriched with Pan-T cell isolation kit II (Miltenyi Biotec). For CD8^+^ T cell AdTr experiments, the CD8α^+^ T Cell isolation kit (Miltenyi Biotec) was used instead. Samples were passed through LS columns on a QuadroMACS separator (Miltenyi Biotec) in MACS buffer (2% FBS + 2 mM EDTA)

### Cell harvest and fluorescence staining for flow cytometry and cell sorting.

Spleens and LNs were harvested and passed through a 40 μm cell strainer (Corning), followed by lysis of RBCs via 2-minute incubation with ammonium chloride-potassium (ACK) lysis buffer (Quality Biological). After magnetic enrichment for T cells, approximately 2 × 10^7^ cells were stained with a fixable live/dead stain (Invitrogen), followed by tetramer staining. Tetramer staining was performed for 35–45 minutes at room temperature with PE- and APC-conjugated OVA_257-264_ (SIINFEKL):H-2K^b^ tetramers (NIH Tetramer Core Facility). The cells were then stained for extracellular antibodies for 15–20 minutes at 4°C. Samples were fixed with the Invitrogen Fix/Perm buffer kit according to the manufacturer’s instruction. Finally, fixed and permeabilized samples were stained for intracellular markers overnight. For phenotypic analysis, samples were acquired via flow cytometry after fixation and intracellular staining. For cell sorting, samples were sorted into RPMI after extracellular staining.

### In vitro stimulation and staining for IFN-γ and TNF-α.

Splenocyte stimulators from 2W-OVA.F1 mice were treated with ACK lysing buffer (Quality Biological), followed by 30-minute incubation with anti-CD90.2 (53-2.1, BD Biosciences) to deplete T cells. Labeled T cells were depleted with 2 consecutive 35-minute incubations with rabbit complement (Cedarlane) at 37°C and then incubated overnight with 20 μg/mL LPS. In total, 1 × 10^6^ responder cells (Pan-T enriched splenocytes) were plated with 0.5 × 10^6^ stimulators (T-depleted APC’s) in triplicate in a 96-well plate (Corning) and incubated at 37°C overnight. Next, Golgi Plug (BD Biosciences) was added at 1:1,000 and incubated for an additional 6 hours at 37°C. Live/Dead and extracellular staining were performed for 10 and 15 minutes (respectively) on ice, and cells were then fixed with BD Cytofix/Cytoperm according to the manufacturer’s instruction (BD Biosciences). Finally, cells were stained for intracellular IFN-γ and TNF-α and acquired via flow cytometry.

### Flow cytometry and cell sorting acquisition and analysis.

Flow cytometry samples for phenotypic panels and in vitro cytokine stimulation assays were acquired on a Cytek Aurora flow cytometer (5 lasers, 16UV-16V-14B-10YG-8R). For cell sorting, samples were acquired and sorted on a BD Aria II 4-15 (70 μm nozzle), BD Aria Fusion 5-18 (70 μm nozzle), or the Invitrogen Bigfoot (100 μm nozzle). The associated software for each cytometer is as follows: Aurora is Cytek SpectroFlo, Aria, and Aria Fusion are BD FACSDiva, and Bigfoot is Invitrogen Sasquatch Software (SQS). Data were analyzed and visualized with FlowJo software v10.8.1 (FlowJo).

### Fluorescent antibodies for flow cytometry and cell sorting.

Fluorochrome-conjugated antibodies were used to select and sort cell subsets, analyze T cell phenotypes, and determine cytokine production. The following antibodies were used in this study, separated by manufacturer (clone is indicated in parentheses). BioLegend: Ki67-PacificBlue (16A8), CD62L-BV510 (MEL-14), CD73-BV605 (TY/11.8), CD44-FITC (IM7), PD1-PEDazzle594 (RMP1-30), TIGIT-PECy7 (1G9), LAG3-BV785 (C9B7W), IFN-γ (XMG1.2), TNF-α–PECy7 (MP6-XT22), SATB1–Alexa Fluor 594 (O96C6), TIM3-APC/Fire750 (B8.2C12), OX40-BV711 (OX-86), OX40L-PECy7 (RM134L), Tim3-PerCP/Cy5.5 (B8.2C12), CD8-FITC (53-6.7), CD90.2-PECy7 (30-H12), CD90.2-PerCP/Cy5 (53-2.1), CD4-APCCy7 (RM4-5). BD Biosciences: CD90.2-BUV395 (53-2.1), CD4-BUV496 (GK1.5), CD19-BUV661 (1D3), CD11c-BUV661 (N418), F4/80-BUV661 (T45-2342), NK1.1-BUV661 (PK136), TER119-BUV661 (TER-119), CD127-BUV737 (SB/199), CD8-BUV805 (53-6.7), FR4-BV421 (12A5), CTLA4-APCR700 (UC10-4F10-11), NK1.1-eFluor450 (PK136), Ter-119-eFluor450 (Ter-119), Rorγt-BV650 (Q31-378), CD62L-BV605 (MEL-14). Invitrogen: FoxP3–Alexa Fluor 532 (FJK-16s), CD44-BUV737 (IM7), PD1-SB780 (J43), TOX-eFluor660 (TXRX10), EOMES-PerCP/eFluor710 (Dan11mag), F4/80-eFluor450 (BM8), CD49b-eFluor450 (DX5), CD11c-eFluor450 (N418), PD1-PerCP-e710 (J43). Santa Cruz Biotechnology Inc.: NFATc1–Alexa Fluor 488 (7A6), CD30L–Alexa Fluor 680 (RM153).

### RNA-Seq data collection and processing.

RNA-Seq libraries were generated and amplified according to the SmartSeq2 protocol (66). In total, 200 live cells per sample/subset were sorted into 96-well optical PCR plates (Thomas Scientific) containing 4 μL of lysis buffer at 4°C. cDNA sequencing libraries were generated using Nextera XT DNA Library Prep Kit and Nextera XT Index Kit (Illumina). All libraries were sequenced in the same run on a NovaSeq 6000 in a 150 bp/150 bp paired-end configuration. An average of approximately 55 × 10^6^ paired reads was generated per sample.

### RNA-Seq data and processing.

Raw RNA-Seq reads were trimmed for adapter content and filtered for truncated reads using Cutadapt v3.4 (67). Paired-end reads were aligned using STAR v2.6.1b (68) against the GRCm39 (mm39) reference genome and transcriptome annotations, and nonuniquely mapping reads were removed. Per-sample read counts for each gene were quantified sample using featureCounts v2.0.1 (69).

### ATAC-Seq.

Chromatin profiling was performed by ATAC-Seq as described previously (70, 71). In brief, approximately 3,000–50,000 sorted cells were washed in cold PBS and lysed to isolate intact nuclei. Transposition was performed at 37°C for 30 minutes with the Tagment DNA Enzyme and Buffer kit (Illumina). After purification of the transposed DNA with the MinElute PCR purification kit (Qiagen), material was amplified via PCR for 13–14 cycles with Nextera XT Index primers (Illumina). The final product was purified again via MinElute PCR purification kit (Qiagen). Libraries were sequenced in the same run on a NovaSeq 6000 in a 150 bp/150 bp paired-end configuration. An average of 75 × 10^6^ paired reads was generated per sample.

### ATAC-Seq data and processing.

Raw ATAC-Seq reads were trimmed for adapter content and filtered for truncated reads using Cutadapt v3.4 (67). Paired-end reads were aligned using Bowtie2 v2.2.9 (72) against the GRCm39 (mm39) reference genome. Nonuniquely mapping reads and PCR duplicates were filtered with Bamtools v2.5 and Picard v2.21.8, respectively (73, 74). Peaks corresponding to ATAC-Seq cut sites for each sample were called using Genrich v0.6.1 in ATAC-Seq mode (https://github.com/jardplard/Chong_CD8_Pregnancy/tree/a961b9052a81e00eea80be6e319afecec815ea21). Finally, reproducibly identifiable peaks for each experimental group were identified via ChIP-R v1.2.0 (75).

### Processing of ATAC-Seq peak set for differential accessibility analysis.

Reproducibly identifiable peaks across all experimental groups were merged into a single reference peak set using Bedtools v2.27.1 (76). multiBamSummary v3.5.1 from the Deeptools suite (77) was used to generate per-sample read counts at each peak from the reference peak set. The read counts data were then imported into R v4.1.0, and each peak was assigned to a single gene via nearest TSS using GenomicRanges v1.46.1, ChIPpeakAnno v3.28.1, and the Org.mm.eg.db v3.14.0 genomic annotation object (78, 79).

### Sequencing data analysis and visualization.

After completing data preprocessing as described above, the DESeq v1.34.0 package was used to conduct differential expression/accessibility analysis on sequencing data sets (80). For both RNA-Seq and ATAC-Seq, the threshold for determining differential expression/accessibility was FDR *P*_adj_ < 0.1 and absolute value of log_2_ fold-change > 0.9. In addition to DESeq2, we used current versions of the following packages for analysis and visualization (with description of purpose in parentheses): Viridis and RColorBrewer (color scale creation); Gplots, ggplot2, and ggrepel (graphing data and generating heatmaps); Uwot and VennDiagram (UMAP and Venn graphs, respectively); and Tidyverse suite (data set manipulation).

### ATAC-Seq motif analysis and locus visualization.

Motif analysis was performed by identifying unique and common peak sets between 2 experimental groups (using the reproducible peaks for each group as described above). These peak sets were then analyzed via HOMER de novo motif analysis (81) to search for significantly enriched motifs associated with ATAC-Seq cut sites and annotate these motifs to possible transcription factor targets. Individual loci were visualized by generating bigwig files for each sample and importing them into IGV v2.12.3 (82). A single track for each experimental group was created by summing the read counts of 2 representative samples from each group.

### GSEA.

GSEA software (4.0.3) was downloaded from the Broad Institute (https://www.gsea-msigdb.org/gsea/index.jsp), and preranked GSEA was performed on the selected gene sets in this study. Gene set files were downloaded from the Molecular Signatures Database or prepared manually as gene matrix expression files (.GMX), using DESeq2 on published RNA-Seq data. Ranked gene lists for our transcriptional data were generated from by arranging genes based on the Change Metric (fold change × −log_10_ *P*_adj_) from high to low. The Change Metric combines both significance and intensity of expression changes, while preserving the direction (up- or downregulation) with positive or negative values.

### Pathway analysis.

Two lists of DEGs (or DAPs) were created for each pairwise comparison — 1 for upregulated/opened regions, and 1 for downregulated/closed regions. The ENSEMBL gene IDs of each list were then uploaded to Metascape Pathway analysis (83) to calculate the enrichment and significance of functional gene pathways from Gene Ontology (GO), KEGG, Reactome, or WikiPathways databases (primarily GO).

### Computational resources.

All data preprocessing for both ATAC-Seq and RNA-Seq (adapter trimming, alignment, filtering, generation of read-counts, and peak calling) was performed on the Midway2 high-performance compute cluster, which is maintained by the University of Chicago Research Computing Center.

### Statistics.

Statistical significance analyses were performed using GraphPad Prism version 9.2.0. A sample size of > 5 animals per experiment were chosen to ensure adequate power. Graft survival significance was assessed using a Kaplan-Meier/Mantel-Cox log rank test. *P* < 0.05 were considered to indicate a significant difference. To calculate differences between experimental animals, we used Kruskal-Wallis test (ANOVA) with Dunn’s post hoc test for pairwise multiple comparisons, 1-way ANOVA with Tukey’s post hoc test, or Welch’s unpaired *t* test (specific tests for each subfigure are indicated in the figure legends). *P* < 0.05 was considered statistically significant.

### Study approval.

All animal experiments were approved by the IACUC at the University of Chicago and adhered to the standards of the *Guide for the Care and Use of Laboratory Animals* (National Academies Press, 2011).

### Data availability.

The RNA-Seq and ATAC-Seq data have been deposited as a SuperSeries in the Gene Expression Omnibus (accession code GSE216302). All scripts used for data analysis have been uploaded to GitHub at https://github.com/jardplard/Chong_CD8_Pregnancy/tree/a961b9052a81e00eea80be6e319afecec815ea21 Values for all data points in graphs are reported in the Supporting Data Values file. Additional information and materials will be made available upon request.

### Code availability.

All code was generated based on publicly available software packages; scripts used for data analysis have been uploaded to GitHub at https://github.com/jardplard/Chong_CD8_Pregnancy

## Author contributions

Authorship order was made after discussion between co–first and co–senior authors, with additional recommendation from 3 anonymous faculty at the University of Chicago. JMP designed and performed the experiments and analyzed data, including pipeline design and script writing for ATAC-Seq and RNA-Seq preprocessing and downstream analyses. JMP wrote the manuscript and generated figures, with guidance from ASC and MLA. GH also performed experiments that generated flow cytometry data and analyzed RNA-Seq and ATAC-Seq data, generated figures, and edited the manuscript. DY and ASC conceived the initial project, and DY performed skin and heart transplantations. MM and FG provided supervision on ATAC-Seq and integrated bioinformatics analysis. All authors provided feedback on figure layout and manuscript content.

## Supplementary Material

Supplemental data

Supporting data values

## Version 1 05/21/2024

In-Press Preview

## Version 2 07/08/2024

Electronic publication
