# Supplementary material for: Pregnancy dedifferentiates memory CD8+ T cells into hypofunctional cells with exhaustion-enriched programs
Source: JCI Insight. 2024 May 21;9(13):e176381. doi: 10.1172/jci.insight.176381 (PMC11383355; doi:10.1172/jci.insight.176381)
Supplement: Supplemental data [file jciinsight-9-176381-s150.pdf]

Extended Data Figures

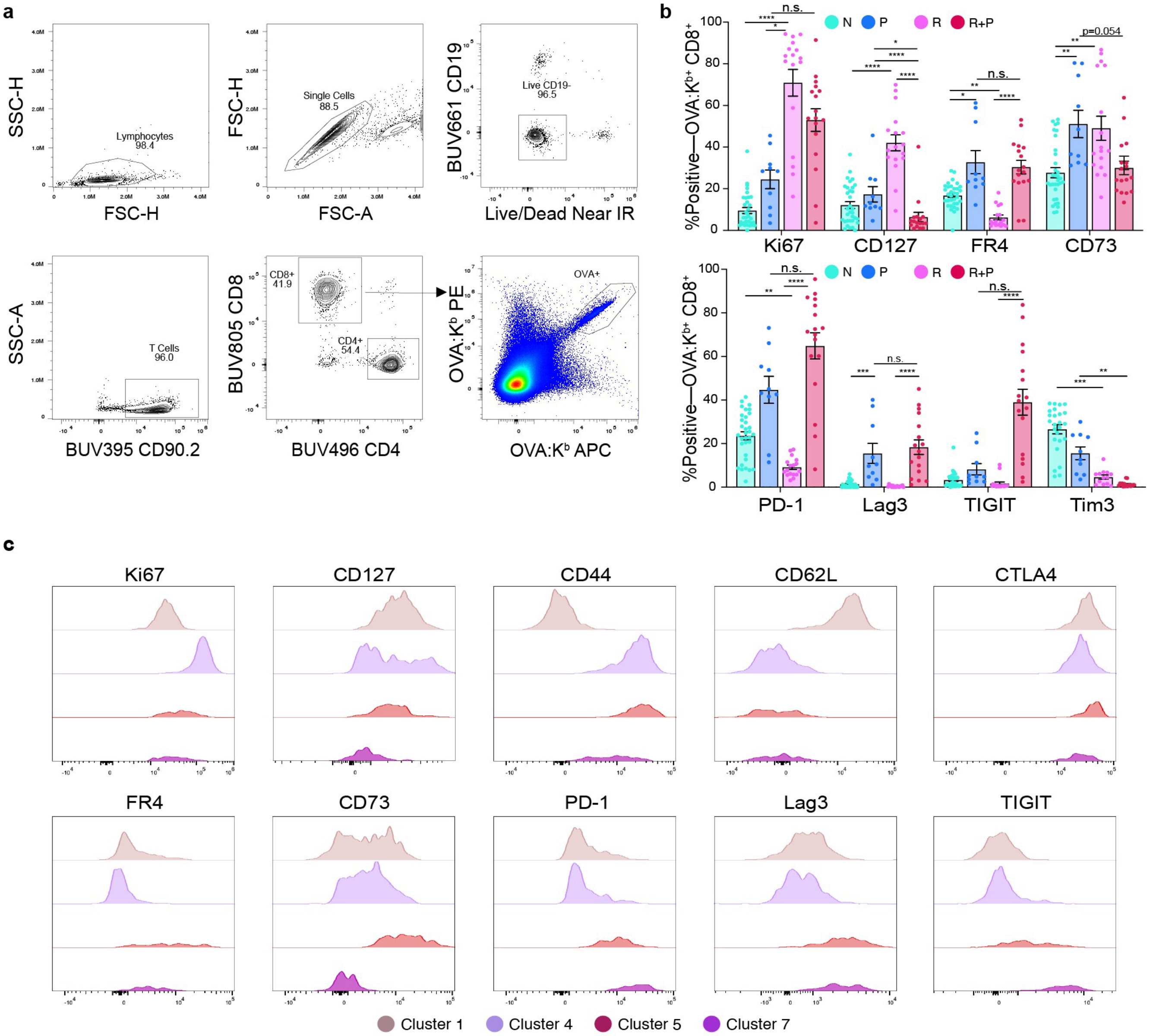

Extended Data Figure 1: Memory and naive OVA-specific T_{FGS} are phenotypically modified by pregnancy. a, Gating strategy for the selection of OVA:K^b-specific CD8$^+$ T cells (T_{FGS}) via spectral flow cytometry. **b**, Bar graph showing percentages of T_{FGS} cells from each experimental group (N: naive; R: D30 post-skin transplant; P or R+P: post-partum day 0-3) expressing phenotypic activation and coinhibitory markers. Data acquired from 2 or more biologically independent experiments, and each dot indicates individual mice; *n* = 10-33 per group. Data represent mean ± SEM. P values were determined by Kruskal-Wallis 1-way ANOVA test with Dunn's post hoc test. ns: not significant; *P<0.05; **P<0.01; ***P<0.001; ****P<0.0001. **c**, Histograms showing phenotypic expression for FlowSOM clusters 1, 4, 5,

and 7. Cluster 1 is predominantly Naive T_{FGS}, cluster 4 is predominantly R T_{FGS}, cluster 5 is shared by P and R+P T_{FGS}, and cluster 7 is unique to R+P T_{FGS}.

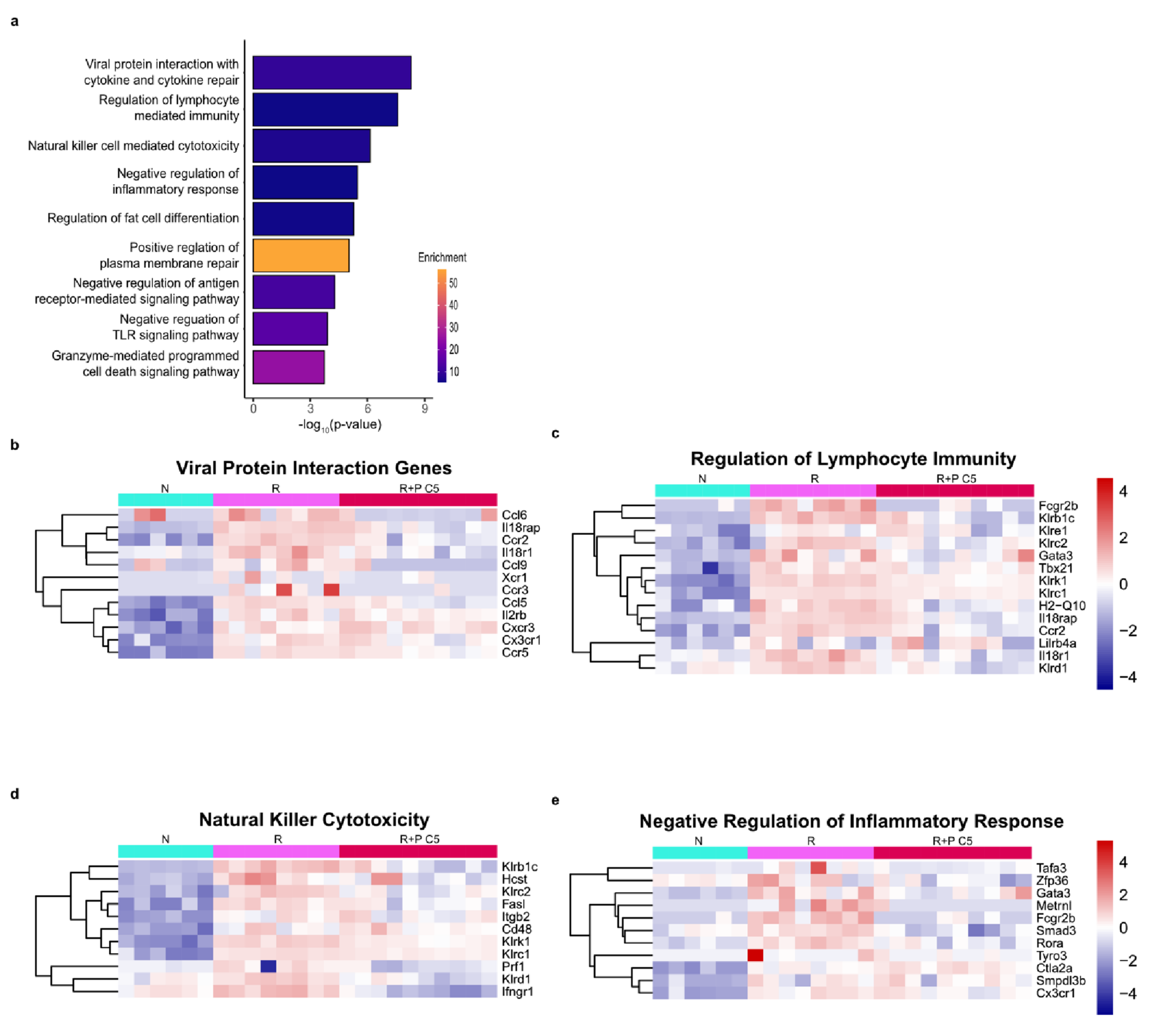

Extended Data Figure 2: Analysis of DEGs from box plots visualizing relative expression of DEGs in each K-Means cluster D from **Fig 3c**. **a**, Metascape pathway analysis of the 362 DEGs. **b-e**, Heatmap of representative genes in the indicated Metascape pathways. Each column indicates individual mice.

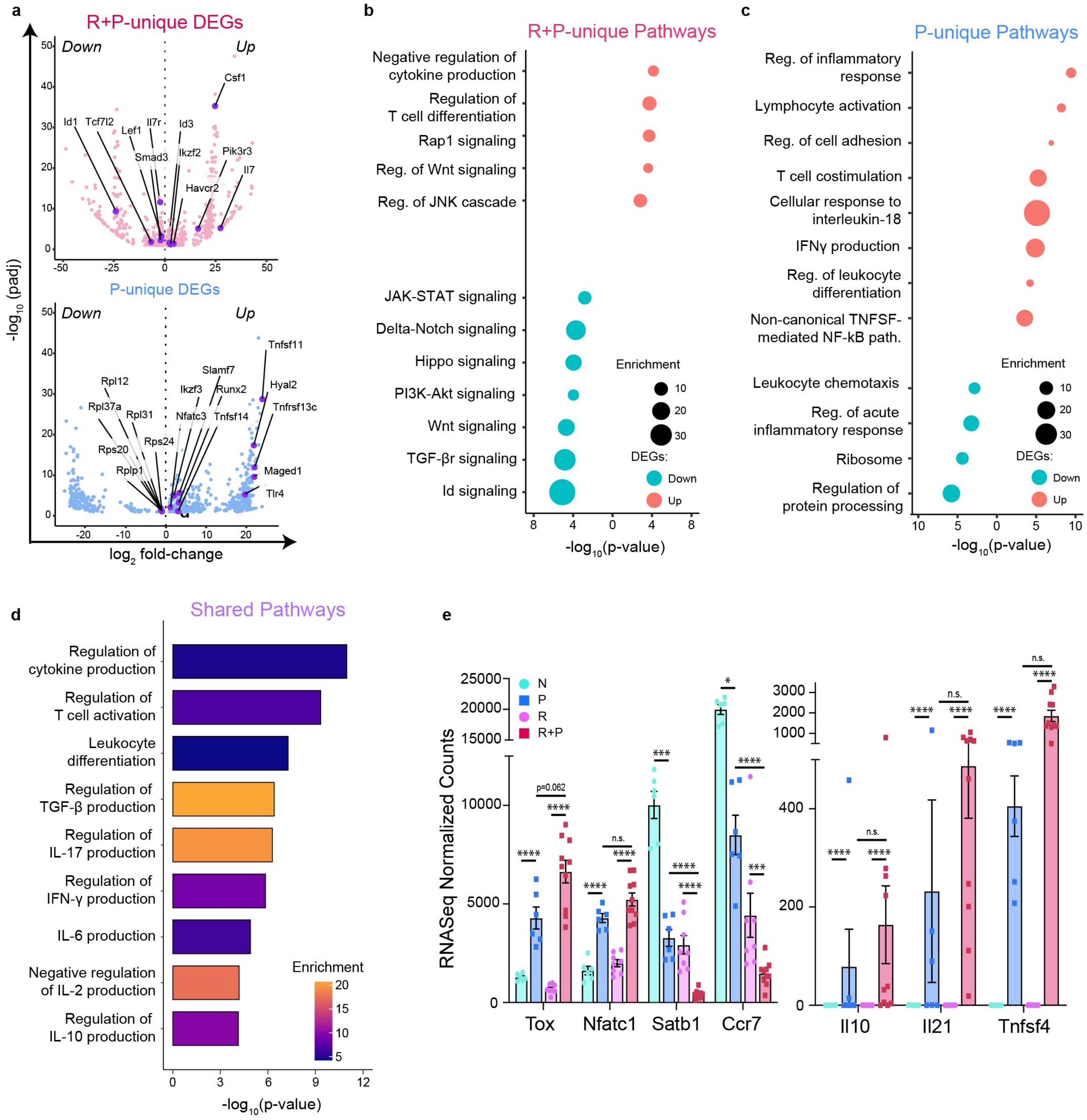

Extended Data Figure 3: Comparison of transcriptional differences between OVA-specific T$_{FGS}$ subsets. a, Volcano plot of DEGs induced in P vs N, and R+P vs R T$_{FGS}$. **b-d**, Metascape pathway analysis for DEGs induced in R+P vs R (**b**), P vs N (**c**) and shared by R+P and P (**d**) T$_{FGS}$. **e,** Normalized RNAseq counts as bar graphs for indicated DEGs. Each dot indicates individual mice. Data acquired from 2 or more biologically independent experiments and represent mean ± SEM. P values were

determined by Kruskal-Wallis 2-way ANOVA test with Dunn’s post hoc test. ns: not significant; *P<0.05; ***P<0.001; ****P<0.0001.

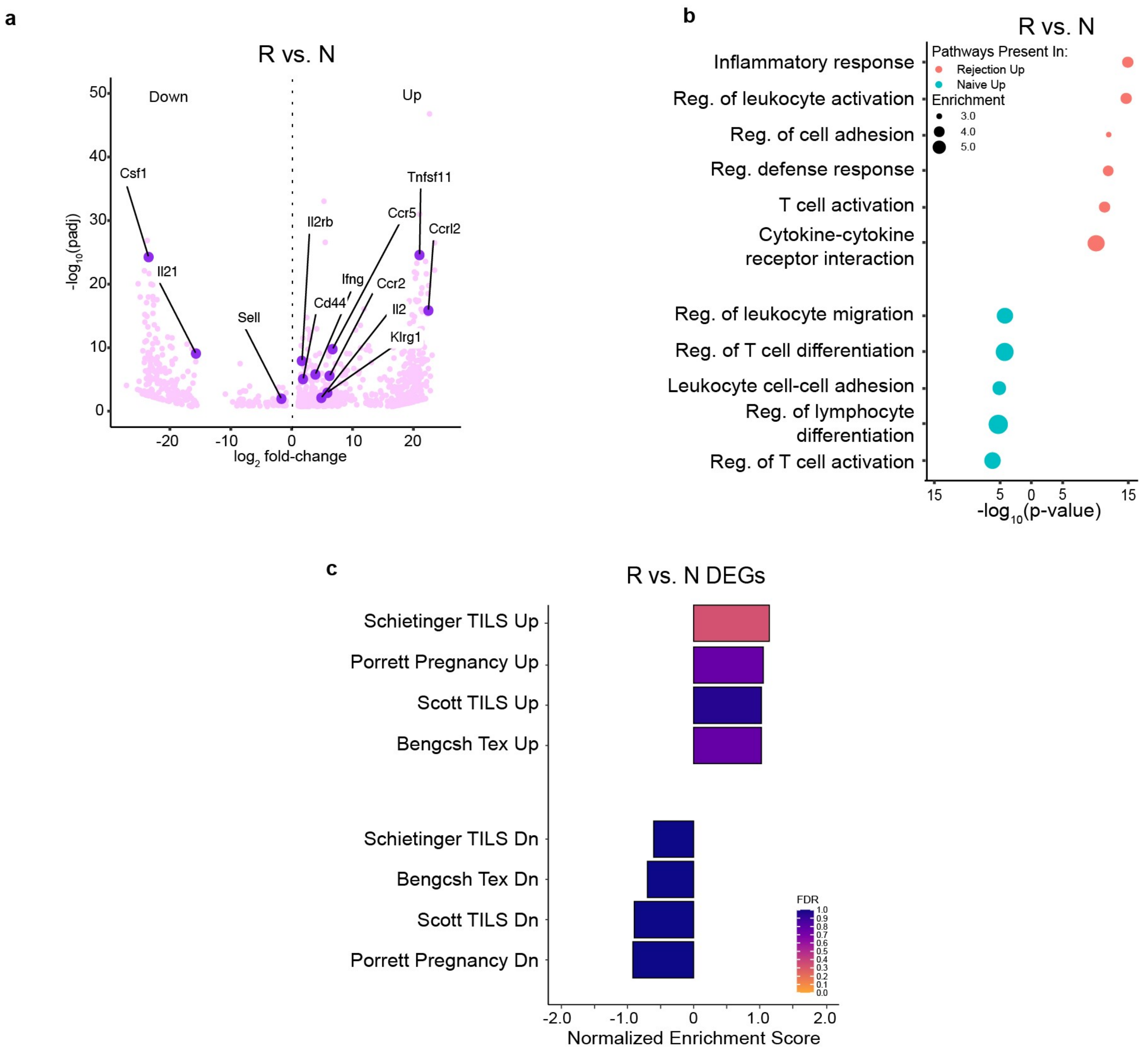

Extended Data Figure 4: Transcriptional differences between OVA-specific T_{FGS} from R vs N are not enriched for exhaustion. a-b, Volcano plot and Metascape pathway analysis of DEGs induced in R vs N, T_{FGS}. c, GSEA analysis comparing DEGs unique to R vs. N to published gene sets of exhaustion (6, 38-40).

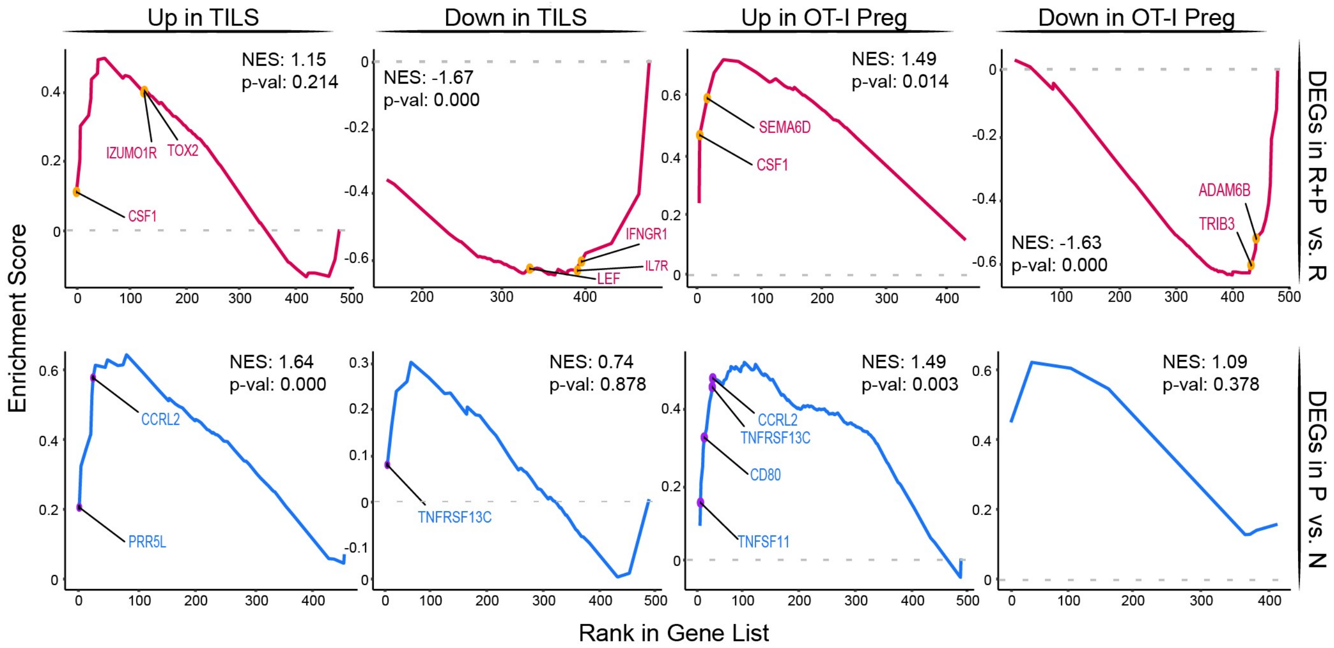

Extended Data Figure 5: GSEA of transcriptional exhaustion by post-partum naïve and memory OVA-specific T_{FGS}. GSEA curves showing enrichment of exhaustion T cell signatures (either up or down regulated) during tumor responses (TILS) and pregnancy (6, 40) in R+P (top row) vs. R and P vs. N (bottom row) DEGs. NES, Normalized Enrichment Score.

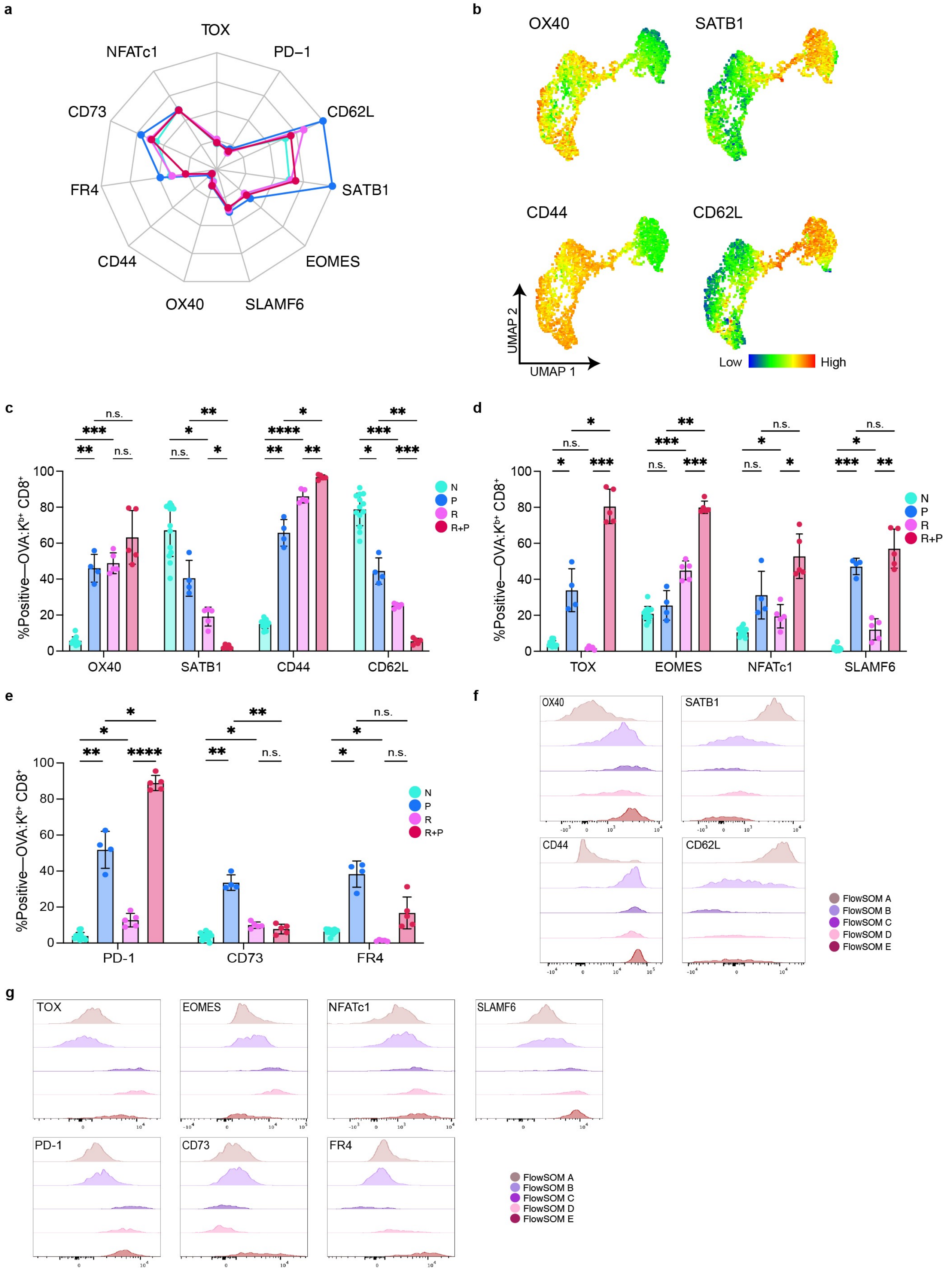

a
TOX
PD−1
CD62L
SATB1
EOMES
SLAMF6
OX40
CD44
FR4
CD73
NFATc1
b
OX40
SATB1
CD44
CD62L
UMAP 2
UMAP 1
Low
High
c
%Positive—OVA:K^b+ CD8$^+$
OX40
SATB1
CD44
CD62L
N
P
R
R+P
n.s.
d
%Positive—OVA:K^b+ CD8$^+$
TOX
EOMES
NFATc1
SLAMF6
N
P
R
R+P
n.s.
e
%Positive—OVA:K^b+ CD8$^+$
PD-1
CD73
FR4
N
P
R
R+P
n.s.
f
OX40
SATB1
CD44
CD62L
FlowSOM A
FlowSOM B
FlowSOM C
FlowSOM D
FlowSOM E
g
TOX
EOMES
NFATc1
SLAMF6
PD-1
CD73
FR4
FlowSOM A
FlowSOM B
FlowSOM C
FlowSOM D
FlowSOM E

Extended Data Figure 6: New phenotypic panel enhances separation of post-partum memory vs. naive OVA-specific T_{FGS}. a, Radar plot showing phenotypic profile of non-OVA:K^b-specific $CD8^+$ T cells from N, P, R and R+P mice. Data are represented as normalized MFI of the highest/lowest MFI for each marker for T_{FGS} and non-T_{FGS} from the 4 experimental groups. **b**, UMAP with heatmap overlays of additional phenotypic markers expressed by T_{FGS} across experimental groups. **c-e,** Bar graphs showing percentages of T_{FGS} cells expressing phenotypic markers of activation and exhaustion. Data acquired from 2 or more biologically independent experiments; *n* = 4-13 per group. Each dot indicates individual mice. Data represent mean ± SEM. P values were determined by Kruskal-Wallis 2-way ANOVA test with Dunn's post hoc test. ns: not significant; *P<0.05; **P<0.01; ***P<0.001; ****P<0.0001. **f-g**, Histograms showing phenotypic expression for FlowSOM clusters from Figure 6c. FlowSOM A is predominantly N T_{FGS}, FlowSOM B is predominantly R T_{FGS}, FlowSOM C+D are predominantly R+P T_{FGS}, and FlowSOM E is unique to P T_{FGS}.

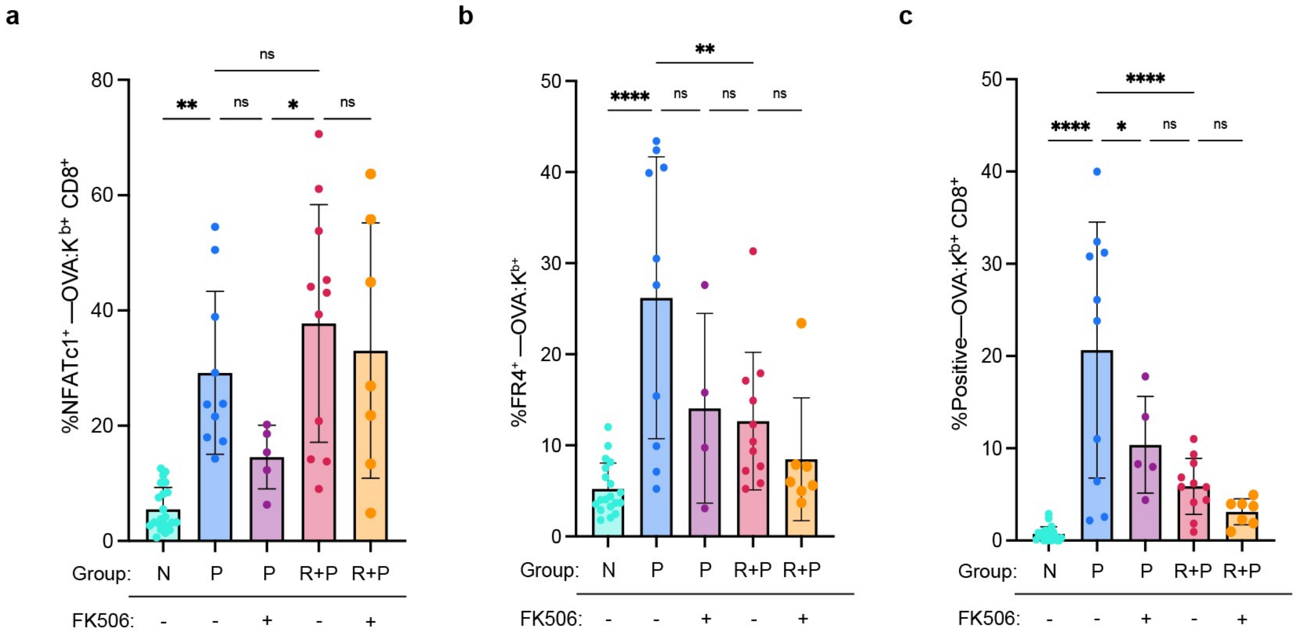

Extended Data Figure 7: Pregnancy-induced phenotypic changes in OVA-specific R+P T_{FGS} resist NFAT inhibition. a-c, Bar graphs showing percentages of T_{FGS} cells expressing additional phenotypic markers of activation and exhaustion from dams treated with FK506. Data acquired from 2 or more biologically independent experiments; *n* = 4-13 per group. Data represent mean ± SEM. P values were determined by 1-way ANOVA. Each dot indicates individual mice. ns: not significant; *P<0.05; **P<0.01; ****P<0.0001.

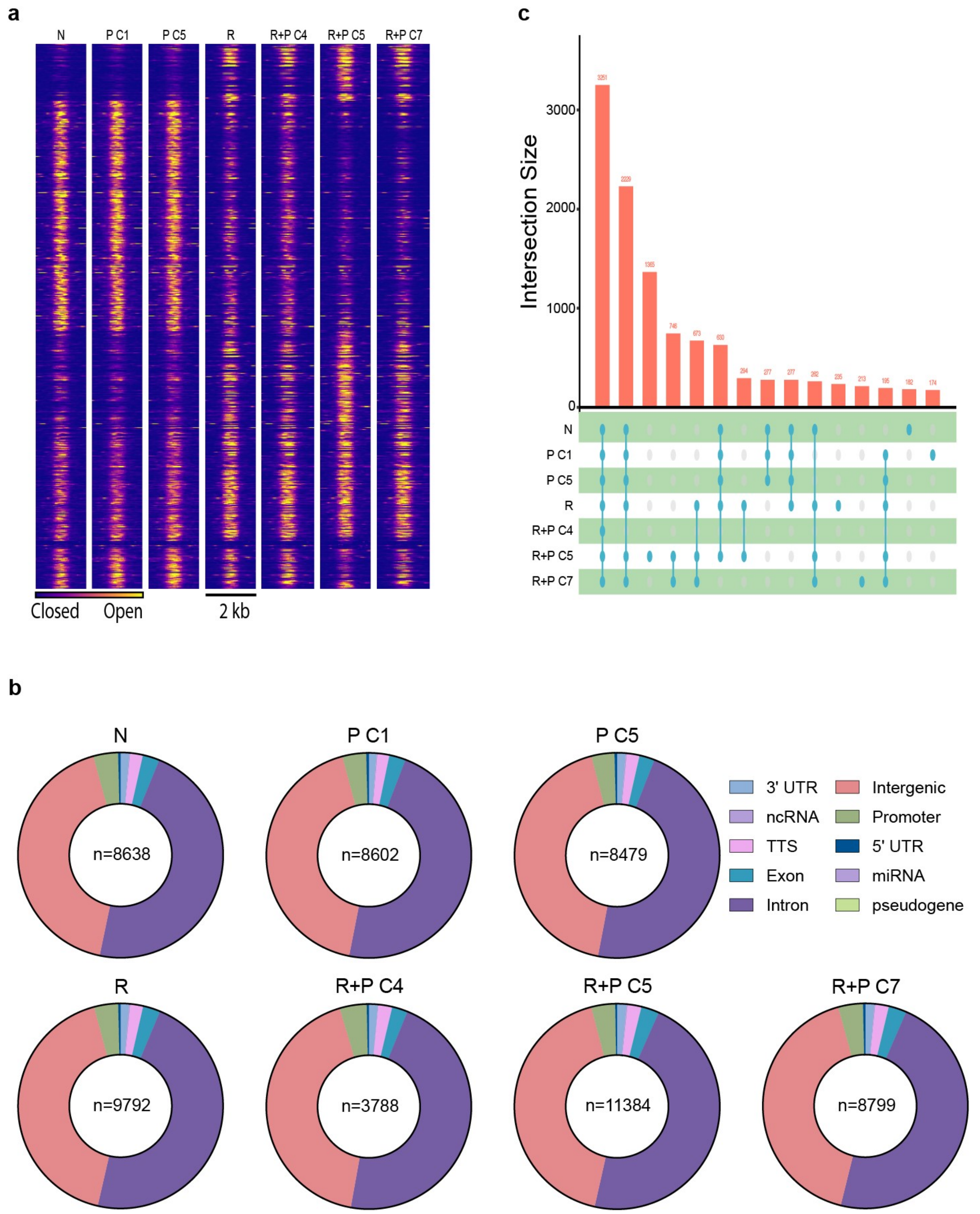

Extended Data Figure 8: Peak distribution and visualization of ATAC-Seq dataset for 7 flowsorted OVA-specific T_{FGS} from N, P, R and R+P groups. a, Chromatin accessibility heatmaps to further

visualize global differences between T_{FGS} subsets. **b**, Pie charts showing the genomic distribution of reproducible ATAC-Seq peaks identified for each T_{FGS} subset. **c,** Upset plot showing the total number of reproducible peaks shared by various combinations of T_{FGS} subsets. This graph serves the same purpose as a Venn diagram but maintains visual proportionality even when comparing across multiple groups.

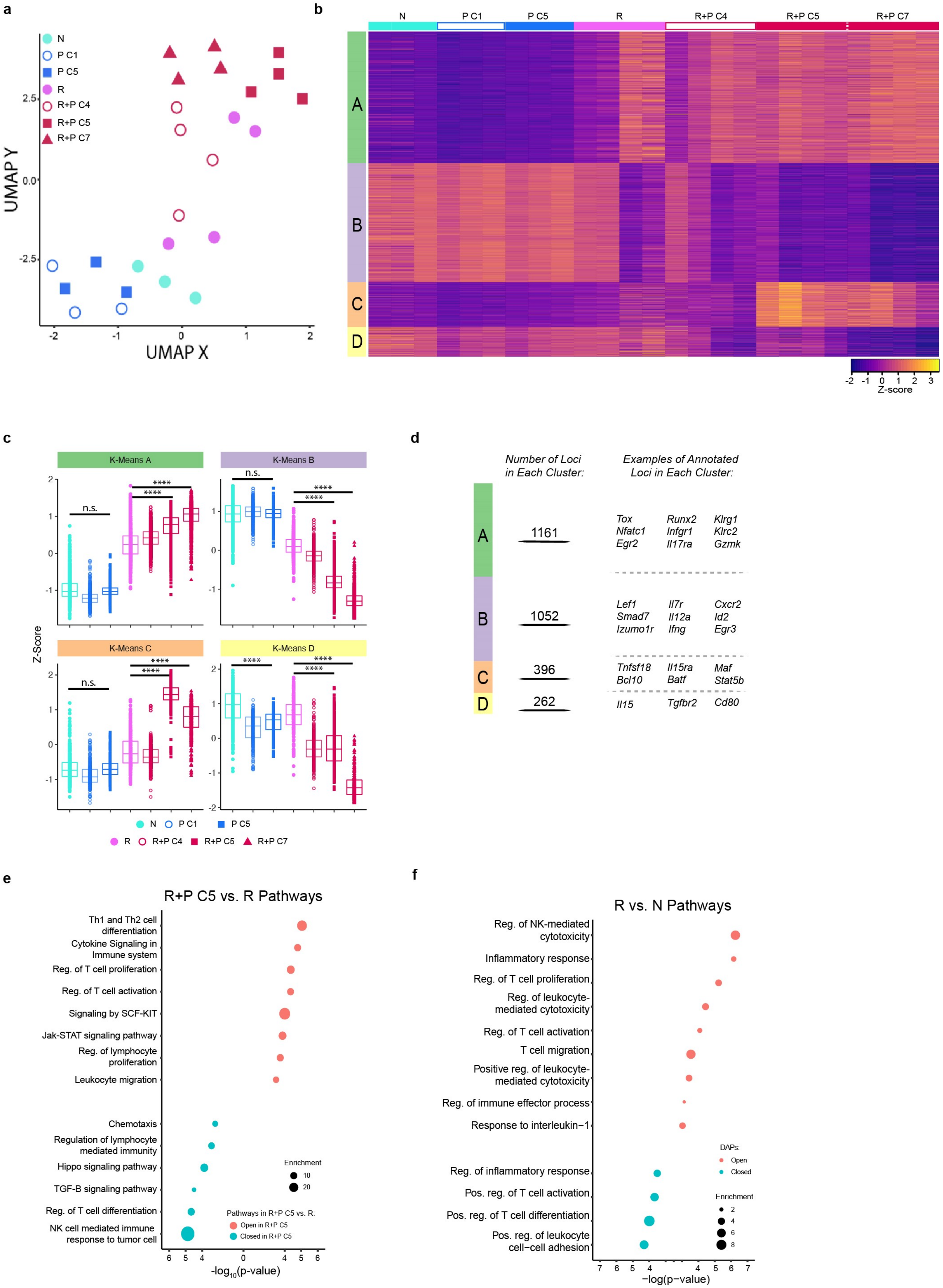
a
N
P C1
P C5
R
R+P C4
R+P C5
R+P C7
UMAP Y
UMAP X
b
N
P C1
P C5
R
R+P C4
R+P C5
R+P C7
A
B
C
D
Z-score
c
K-Means A
K-Means B
K-Means C
K-Means D
Z-Score
n.s.
****
N P C1 P C5
R R+P C4 R+P C5 R+P C7
d
Number of Loci in Each Cluster:
Examples of Annotated Loci in Each Cluster:
A 1161 Tox Nfatc1 Egr2 Runx2 Infgr1 Il17ra Klrg1 Klrc2 Gzmk
B 1052 Lef1 Smad7 Izumo1r Il7r Il12a Ifng Cxcr2 Id2 Egr3
C 396 Tnfsf18 Bcl10 Il15ra Batf Maf Stat5b
D 262 Il15 Tgfbr2 Cd80
e
R+P C5 vs. R Pathways
Th1 and Th2 cell differentiation
Cytokine Signaling in Immune system
Reg. of T cell proliferation
Reg. of T cell activation
Signaling by SCF-KIT
Jak-STAT signaling pathway
Reg. of lymphocyte proliferation
Leukocyte migration
Chemotaxis
Regulation of lymphocyte mediated immunity
Hippo signaling pathway
TGF-B signaling pathway
Reg. of T cell differentiation
NK cell mediated immune response to tumor cell
Enrichment
Pathways in R+P C5 vs. R:
Open in R+P C5
Closed in R+P C5
-log10(p-value)
f
R vs. N Pathways
Reg. of NK-mediated cytotoxicity
Inflammatory response
Reg. of T cell proliferation
Reg. of leukocyte-mediated cytotoxicity
Reg. of T cell activation
T cell migration
Positive reg. of leukocyte-mediated cytotoxicity
Reg. of immune effector process
Response to interleukin−1
Reg. of inflammatory response
Pos. reg. of T cell activation
Pos. reg. of T cell differentiation
Pos. reg. of leukocyte cell−cell adhesion
DAPs:
Open
Closed
Enrichment
−log(p−value)

Extended Data Figure 9: Comparison of pregnancy-induced chromatin remodeling in memory vs. naive OVA-specific T_{FGS}. a-b, UMAP and row-normalized ATAC-seq accessibility heatmap of the top differentially accessible peaks across all T_{FGS} subsets (from **Fig 2a**), organized by K-means clustering (colored bar on left indicate 4 clusters A-D). **c**, Box plots visualizing chromatin accessibility at DEGs unique to each K-Means cluster identified. Data acquired from 2 or more biologically independent experiments. Each dot (**a,c**) or column (**b**) indicates individual mice. P values were determined by Welch's 2-tailed t-test. ns: not significant; ****P<0.0001. **d**, Number of loci and examples of annotated loci for each K-Means cluster A-D. **e-f**, Metascape pathway/gene ontology analysis for differentially accessible peaks in R+P vs. R T_{FGS} (**e**), and R vs. N T_{FGS} (**f**).

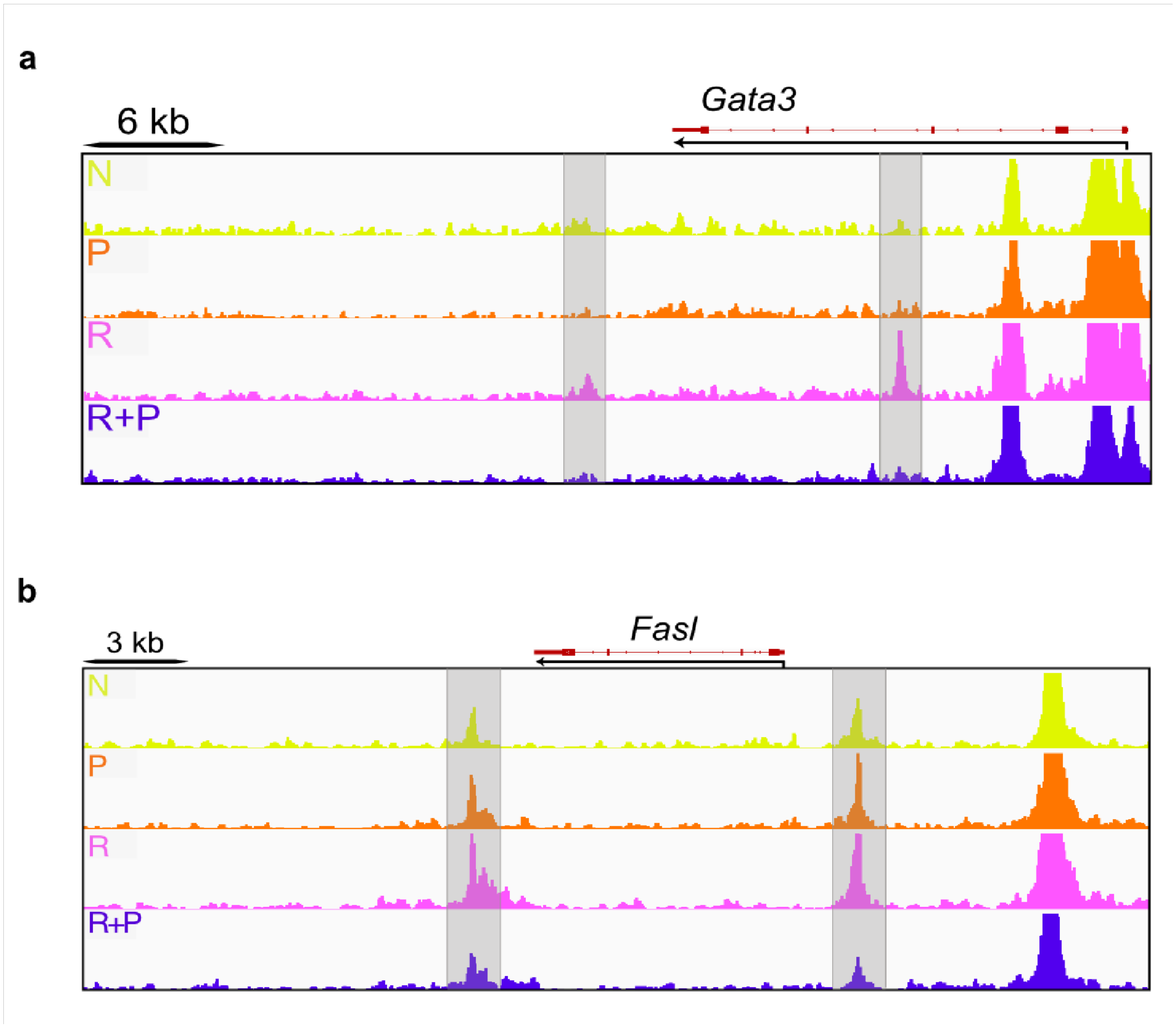

Extended Data Figure 10: ATAC-Seq tracks at the (**a**) *Gata3* and (**b**) *Fasl* loci. Peaks uniquely induced in R T_{FGS} are highlighted in gray.

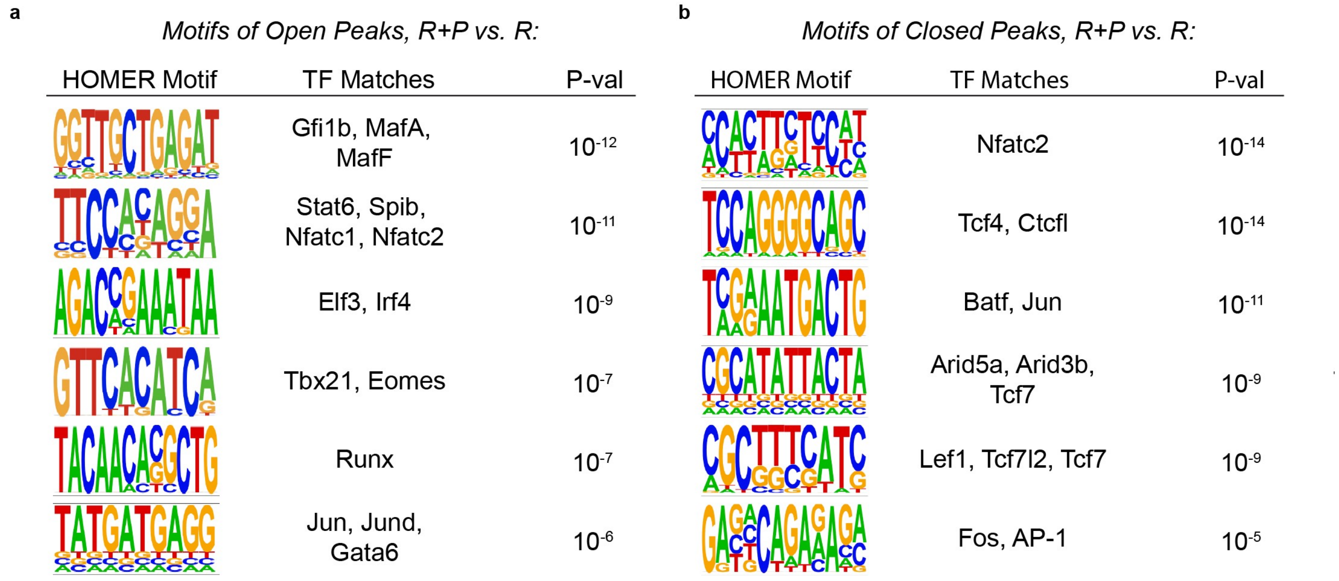

Extended Data Figure 11: HOMER de-novo analysis of nucleotide motifs associated with transcription factor binding in post-partum memory OVA-specific T_{FGS}. Transcription factor binding motifs that were significantly enriched in reproducible ATAC peaks opening (**a**) or closing (**b**) in R+P vs. R T_{FGS}. P values were determined by Homer de novo motif analysis.

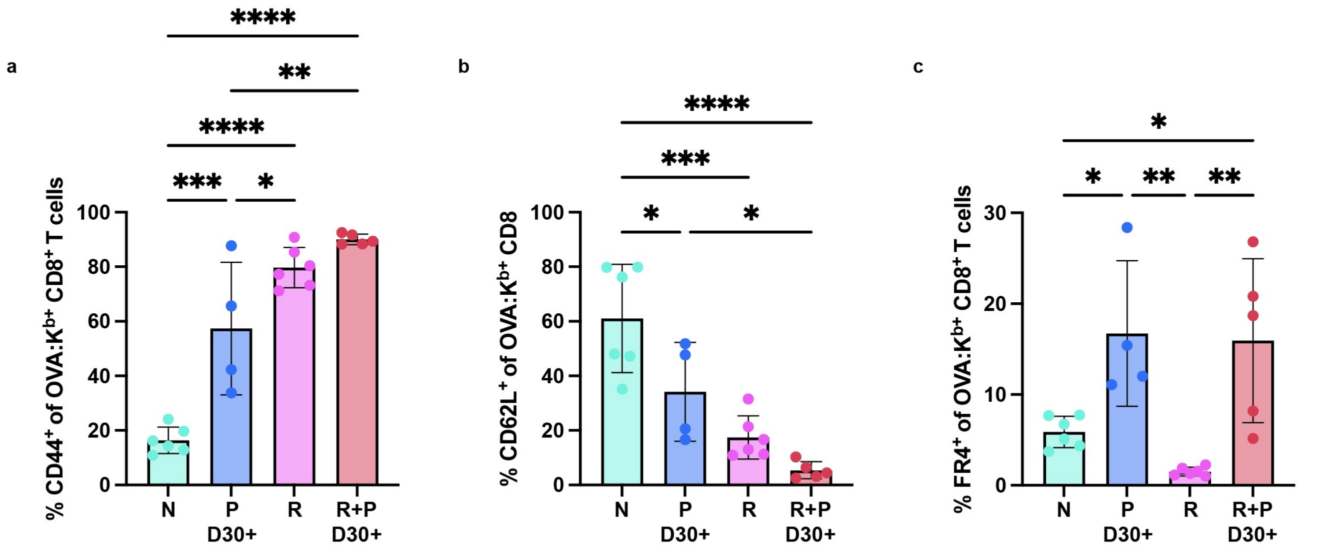

Extended Data Figure 12: Pregnancy induces persistent exhausted phenotype in post-partum memory OVA-specific T_{FGS}. a-c, Bar graphs showing percentage of OVA-specific $\mathbf{T_{FGS}}$ from P and R+P (both at post-partum day 30), Naive (N) or R (day 30-60 post-skin transplant) expressing additional phenotypic markers. Data acquired from 2 or more biologically independent experiments; n=4-6 per group. Each dot indicates individual mice, and data represent mean ± SEM. P values determined by one-way ANOVA. ns: not significant; *P<0.05; **P<0.01; ****P<0.0001.

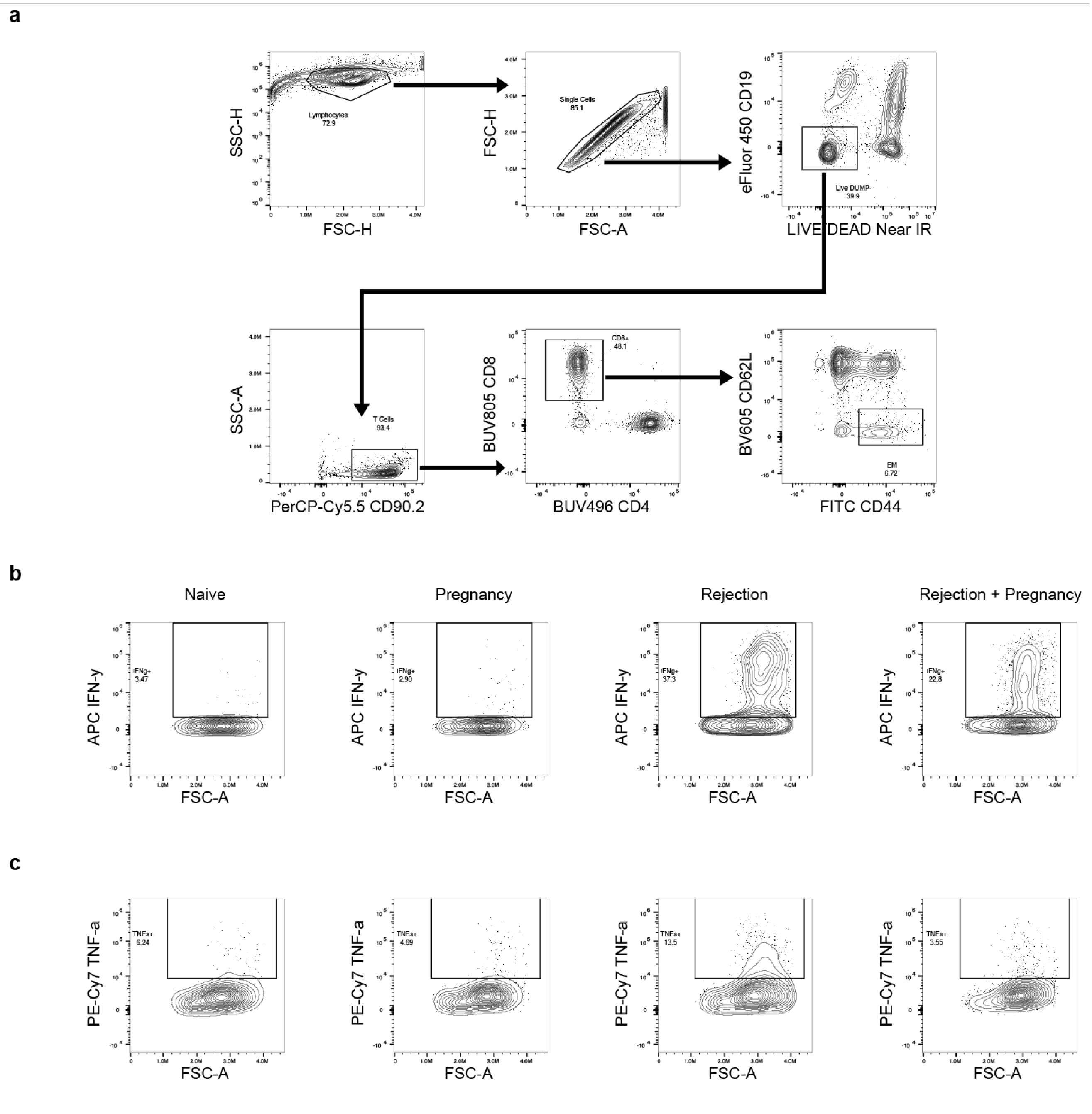

Extended Data Figure 13: Pregnancy reduces effector capacity of post-partum memory and naive OVA-specific T_{FGS}. a, Gating strategy for the selection of bulk $CD62L^{low}CD44^{+}$ $CD8^{+}$ T cells for analysis of intracellular IFN-γ^{+} and TNF-α^{+} by spectral flow cytometry. **b-c,** Representative plots of IFNγ^{+} (**b**) and TNF-α^{+} (**c**) T cells stimulated with 2W-OVA.F1 T cell-depleted splenocytes, for each experimental group (N, R, as well as P and R+P at post-partum day 0-3).

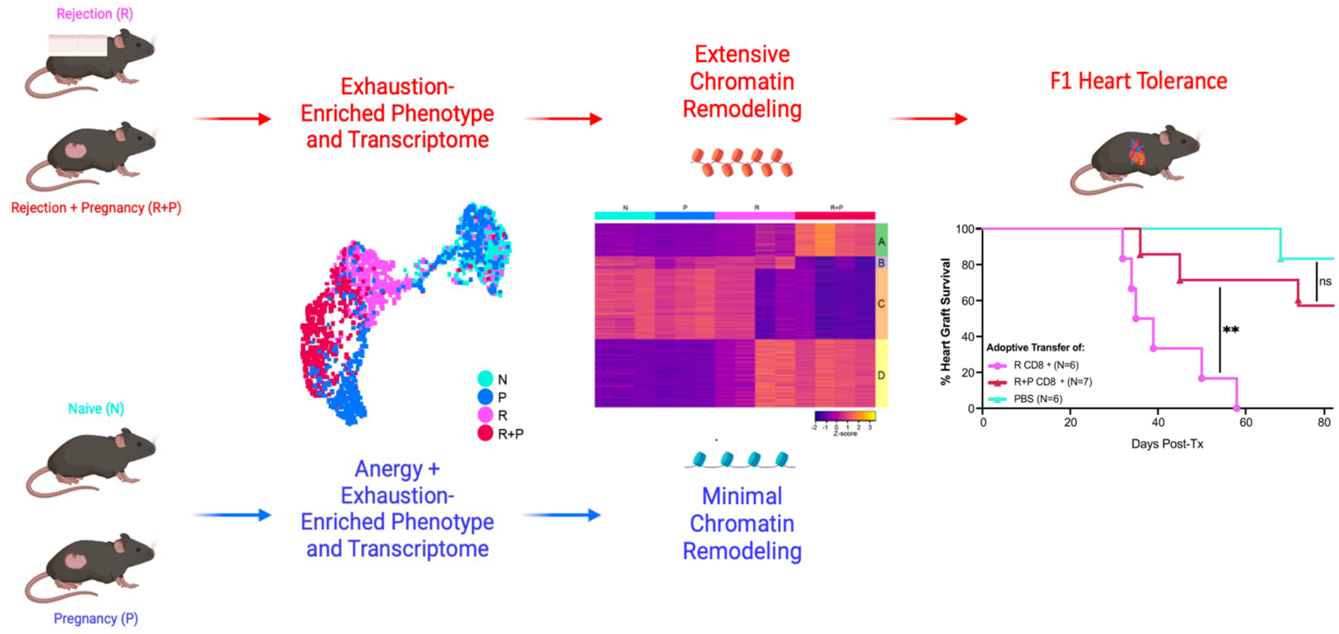

Extended Data Figure 14: Pregnancy adaptively utilizes multiple distinct mechanisms to induce hypofunction in memory vs naive T_{FGS}. Graphical abstract. Pregnancy induces hypofunction in memory R+P T_{FGS} that is associated with phenotypic and transcriptional exhaustion, partial reversal of the memory transcriptome, chromatin remodeling of exhaustion loci, and restored susceptibility to costimulation blockade-mediated acceptance of fetus-matched heart grafts.

Panel 1			Panel 2	
Fluorophore	Marker		Fluorophore	Marker
BUV395	CD90.2		BUV395	CD90.2
BUV496	CD4		Live/Dead Blue	Live/Dead
BUV661	Dump		BUV496	CD4
BUV737	CD127		BUV661	Dump
BUV805	CD8		BUV737	CD44
BV421	FR4		BUV805	CD8
BV450 (PacBlue)	Ki67		BV421	FR4
BV510	CD62L		BV450 (PacBlue)	SLAMF6
BV605	CD73		BV510	CD62L
BV785	LAG3		BV605	CD73
FITC	CD44		BV650	RORγT
AlexaFluor 532	Foxp3		BV711	OX40
PerCP-Cy5.5	Tim3		SB780	PD-1
PE	OVA:Kb		AlexaFluor 488	NFATc1
PE-Dazzle	PD-1		AlexaFluor 532	FOXP3
Pe-Cy7	TIGIT		PerCP-e710	EOMES
APC	OVA:Kb		PE	OVA:Kb
APC-R700	CTLA4		AlexaFluor 594	SATB1
Live/Dead NIR	Live/Dead		Pe-Cy7	OX40L
			APC	OVA:Kb
			eFluor660	TOX
			AlexaFluor 680	CD30
			APC-Cy7 (Fire 750)	TIM3

Supplementary Table 1. Spectral flow panel for Fig 1/Extended Data Figure 1 (Panel 1) and Figure 5/Extended Data Figure 6 (Panel 2).

Experimental Group	Replicate	# of Cells Transferred (in millions)	Day Rejected
PBS	#521	n/a	90
PBS	#522	n/a	90
PBS	#523	n/a	68
PBS	#361	n/a	85
PBS	#362	n/a	85
PBS	#363	n/a	85
Average ± SEM			83.8 ± 4.3
R	#2265	6.1	34
R	#2268	6.3	35
R	#644	5.7	58
R	#646	4.5	39
R	#976	7.6	50
R	#977	5.0	32
Average ± SEM		5.7 ± 0.45	41.3 ± 4.0
R+P	#2242	8.4	45
R+P	#2239	5.4	91
R+P	#2271	7.7	81
R+P	#2623	12.0	90
R+P	#640	6.4	36
R+P	#2771	5.0	102
R+P	#2982	5.3	72
Average ± SEM		7.2 ± 0.88	73.9 ± 8.1

Supplementary Table 2. Total numbers of adoptively transferred CD8+ T cells from R and R+P, and day of heart allograft rejection.
